# A Self-Healing Crystal That Repairs Multiple Cracks

**DOI:** 10.1021/jacs.4c09334

**Published:** 2024-09-18

**Authors:** Javed R. Pathan, Haripriya Balan, Patrick Commins, Arthi Ravi, Marieh B. Al-Handawi, Ian Cheng-Yi Hou, Panče Naumov, Kana M. Sureshan

**Affiliations:** † School of Chemistry, Indian Institute of Science Education and Research Thiruvananthapuram, Thiruvananthapuram, Vithura 695551, India; ‡ Smart Materials Lab, New York University Abu Dhabi, PO Box 129188, Abu Dhabi 129188, United Arab Emirates; § Center for Smart Engineering Materials, New York University Abu Dhabi, PO Box 129188, Abu Dhabi 129188, United Arab Emirates; ∥ Research Center for Environment and Materials, Macedonian Academy of Sciences and Arts, Bul. Krste Misirkov 2, Skopje MK−1000, Macedonia; ⊥ Molecular Design Institute, Department of Chemistry, New York University, 100 Washington Square East, New York, New York 10003, United States

## Abstract

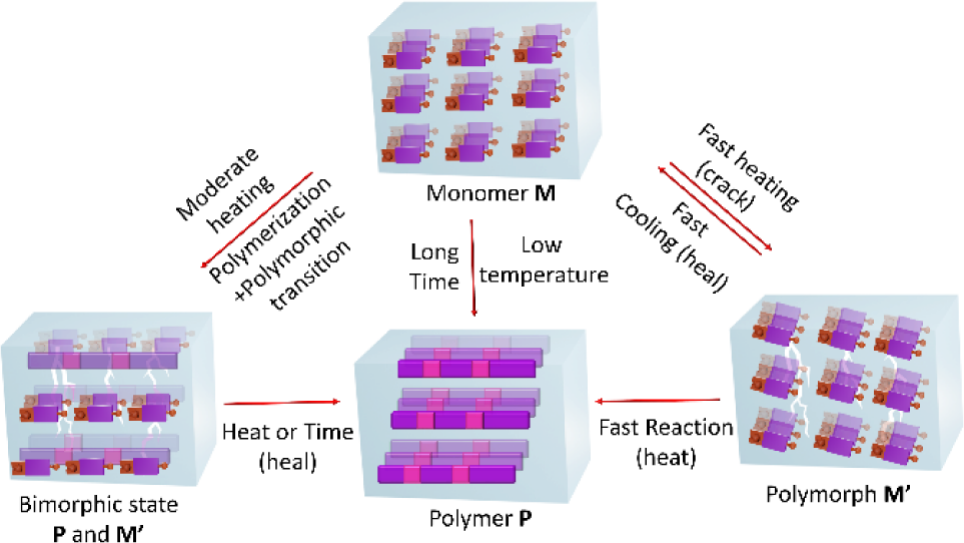

We report both cracking and self-healing in crystals occurring during a thermal phase transition, followed by a topochemical polymerization. A squaramide-based monomer was designed where the azide and alkyne units of adjacent molecules are positioned favorably for a topochemical click reaction. The monomer undergoes spontaneous single-crystal-to-single-crystal (SCSC) polymerization at room temperature via regiospecific 1,3-dipolar cycloaddition, yielding the corresponding triazole-linked polymer in a few days. When heated at 60 °C, the polymerization completes in a SCSC manner in 24 h. Upon continuous heating from room temperature to 110 °C, the monomer crystals develop multiple cracks, and they self-heal immediately. The cracking occurs due to a thermal phase transition, as evidenced by differential scanning calorimetry (DSC). The cracks heal either upon further heating or upon cooling of the crystals due to the topochemical polymerization or reversal of the phase transition, respectively. Increasing the heating rate leads to the formation of longer and wider cracks, which also heal instantaneously. The self-healed crystals retained their integrity and the crystal structure of the self-healed crystals was analyzed by single-crystal X-ray diffraction. The quality of the self-healed crystals and their diffraction ability conform to those of the completely reacted crystals at room temperature or at 60 °C without developing cracks. This work demonstrates a novel mechanism for self-healing of molecular crystals that could expand the horizon of these materials for a plethora of applications.

## Introduction

Self-healing is a typical feature of living systems; they achieve this by transporting the building chemicals to the damage site followed by a series of complex biochemical processes to recover the affected tissue(s). This intriguing property has been replicated with synthetic materials, especially with soft materials, such as polymers^1^ and gels, by exploiting either reversible covalent bond-forming reactions^2−9^ or noncovalent interactions.^10,11^ There is a strong interest in developing self-healing molecular crystals as they could help in our understanding the structural recovery processes at atomic resolution.^12,13^ Crystals, because of their higher order, structural rigidity, and slow diffusion, have been categorized as brittle materials, but with the discovery of flexible^14−18^ and mechanically responsive crystals,^19,20^ this paradigm is being abandoned. Naumov and coworkers rationally designed the first self-healing organic crystal that uses dynamic covalent chemistry, where reversible breaking and reformation of the disulfide bonds between molecules at the damaged surface assists self-healing.^21^ Though the healing efficiency was moderate, this seminal work generated further interest in self-healing crystals. Later, the same group used the reversible boronic ester formation reaction to design self-healing crystals with enhanced self-healing efficiency.^22^ Due to the reversibility of the crucial metathesis reaction and the necessity of material transport, the healing requires several hours of contact and application of a weak compressive force. Tao and coworkers observed that a donor–acceptor cocrystal undergoes reversible polymorphic transition upon heating, and this transition is accompanied by splitting of the crystal along its long axis and rejoining of the fragments.^23^ As the polymorphic transition is slow, self-healing takes several minutes, and the healing involves the rearrangement of molecules and noncovalent interactions.

Later, Reddy and coworkers reported another crystal that shows self-healing via attractive dipole–dipole noncovalent interactions.^24^ A crack introduced by three-point bending undergoes complete self-healing in a fraction of a second after the withdrawal of the bending force; clearly, the healing based on such attractive noncovalent interactions seemingly occur on very short time scales, determined by the time required to make physical contact.^25^ Here, we report the first example of cracking and sudden self-healing of an organic crystal due to a combination of a phase transition and a topochemical reaction. These results demonstrate the mechanistic diversity of the self-healing phenomenon and imply that it is more general and occurs even in molecular crystals undergoing chemical reactions.

## Results and Discussion

### Design and Preparation of the Squaramide Monomer **M**

**Figure 1:**
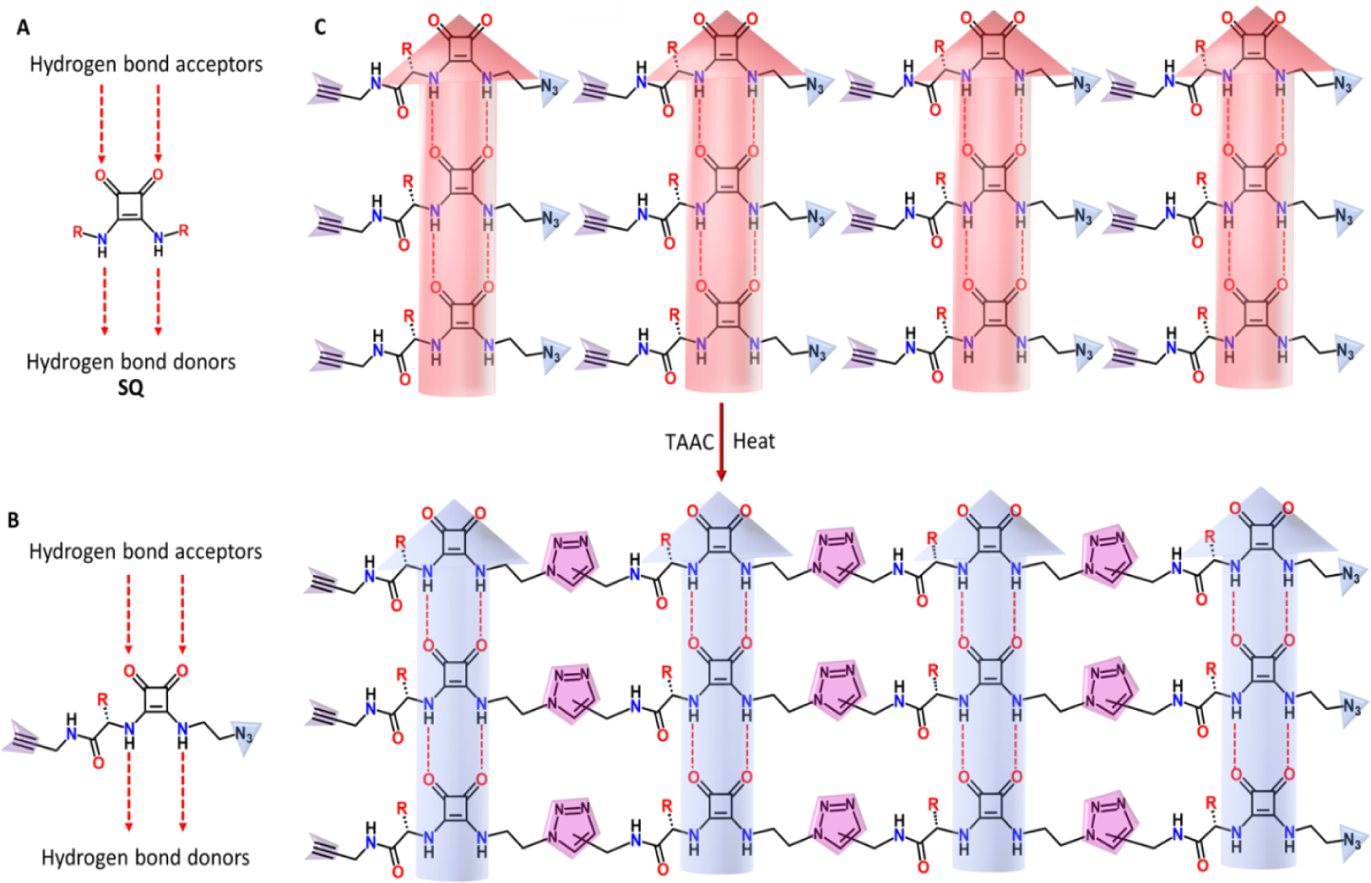
Schematic representation of the self-assembly and topochemical polymerization of squaramide-based monomer. (A) Hydrogen bonding pattern in squaramide. (B) General chemical structure of a squaramide (**SQ**) monomer depicting the hydrogen bonding synthons. (C) Schematic representation showing a squaramide monomer undergoing a topochemical reaction.

**Figure 2:**
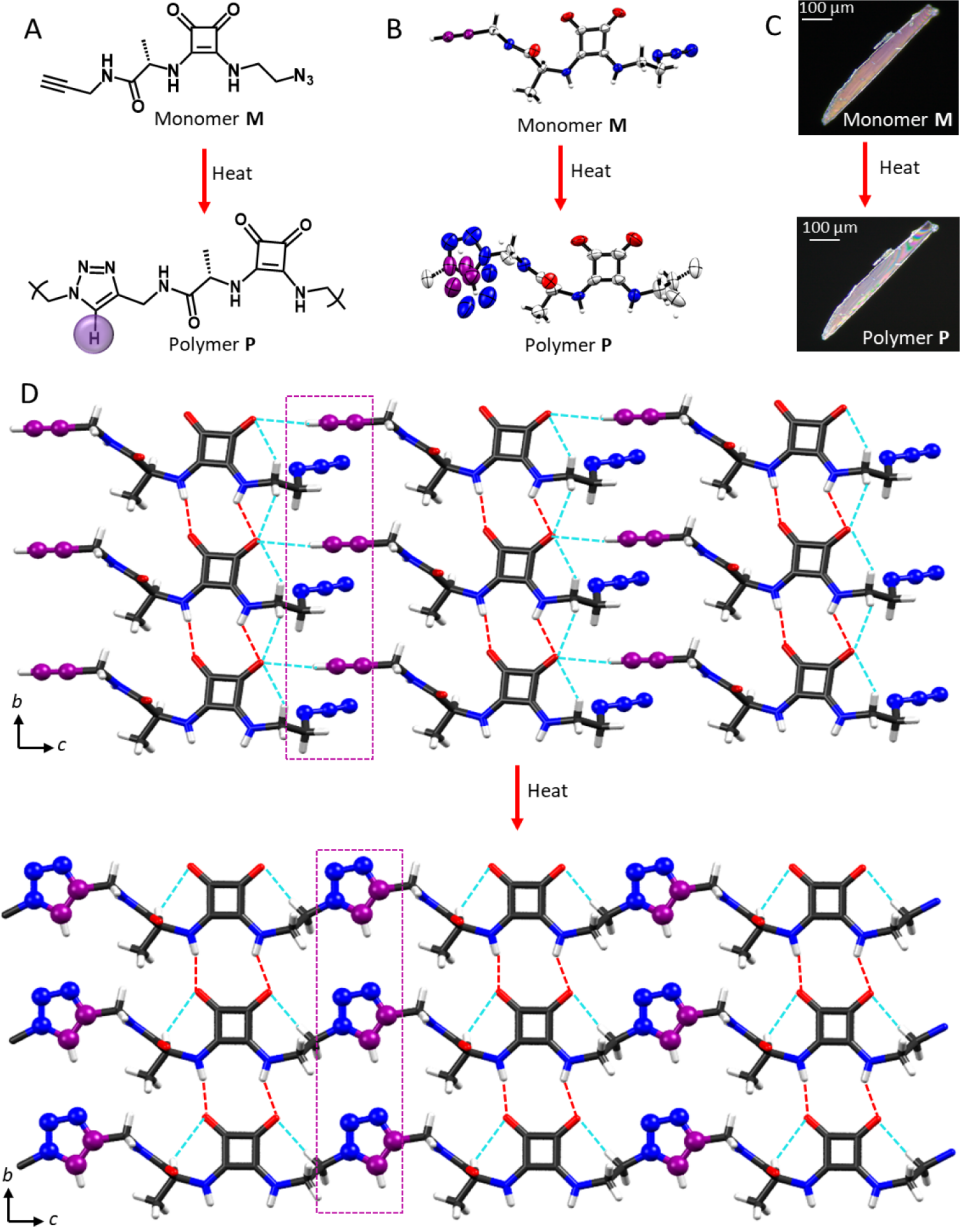
Chemical structure, crystal habit, and crystal structure of the monomer, **M** and the polymer, **P**. (A) Chemical reaction of **M** to form **P**. (B) ORTEP-style diagram of the monomer and the polymer, plotted at 50% probability. (C) Microscopic image of a typical habit of the monomer and polymer crystal. (D) Crystal packing of the monomer and the resulting polymer. The monomer molecules, stacked as hydrogen-bonded columns along the *b* axis, are head-to-tail arranged along the *c* axis with their azide and alkyne groups favorably positioned for a topochemical cycloaddition reaction. Red and cyan dotted lines represent NH···O and CH···O hydrogen bonding.

Our interest in the synthesis of crystalline chiral polymers via topochemical azide–alkyne cycloaddition (TAAC) polymerization prompted us to design a monomer of general structure **SQ** having a squaramide unit for biasing the packing (Figure 1A), a chiral amino acid that imparts chirality, and complementary reactive groups at its termini for polymerization, viz., azide and alkyne groups (Figure 1B). Based on the conserved crystal packing of squaramide derivatives,^26,27^ such a monomer is expected to pack with proximally placed azide and alkyne units in its crystals, and the crystals are expected to undergo TAAC polymerization (Figure 1C). We synthesized the monomer **M** (Figure 2A) by adopting standard peptide synthetic protocols (Section SI2), and we crystallized it from a 10:1 (v/v) mixture of methanol and dichloromethane by slow evaporation (Figure 2). We determined the crystal structure of the plate-like crystals of the monomer **M** by using single-crystal X-ray diffraction. The compound crystallizes in the triclinic (*P*1) space group with one molecule in the asymmetric unit (Figure 2B). The molecule adopts a conformation in which the terminal azidomethylene and propargylamide units point to opposite directions from the squaramide plane. The amide and squaramide planes are at an angle of 73°. Two intermolecular NH···O hydrogen bonds between adjacent squaramide units stack the monomer molecules as a column along the *b* axis, and the stacking is further stabilized by CH···O hydrogen bonds (Figure 2D). NH···O hydrogen bonds between the propargyl amide chain connect the molecules along the *a* axis, and additional CH···O and CH···π hydrogen bonds assist the molecular organization (Figure 2D). The molecules of the monomer are laterally connected in a head-to-tail fashion and interdigitated along the *c* axis by utilizing CH···O hydrogen bonding. This interdigitation places the azide and alkyne units of adjacent molecules at proximity (average distance, 3.5 Å) and in an antiparallel fashion, similar to the transition state anticipated for the cycloaddition reaction to form 1,4-triazolyl-linked polymer. The short distance between the termini of the azide and alkyne units suggests the possibility of a topochemical polymerization reaction. A comparison of the experimental PXRD profile of the bulk crystals with the PXRD profile simulated from the crystal structure confirmed the phase purity of the crystals (Figure S1).

### Spontaneous and Slow Single-Crystal-to-Single-Crystal Polymerization at Room Temperature

**Figure 3:**
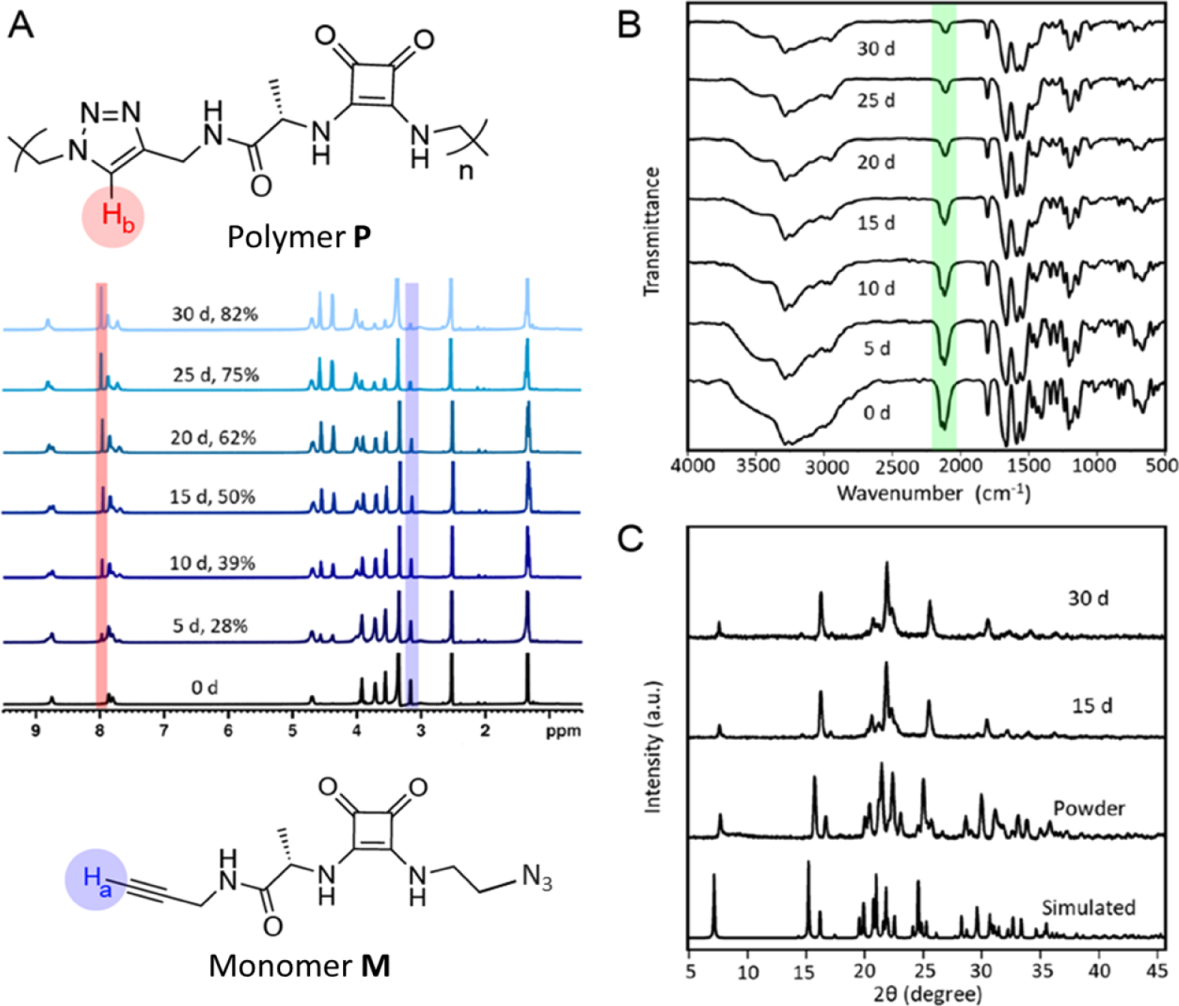
Reactivity of the squaramide monomer **M**. (A) Time-dependent ^1^H NMR analysis of **M** converting into **P**. The alkynyl proton, H_a_, and the triazolyl proton, H_b_, are highlighted in blue and red, respectively. (B) FT-IR spectra showing gradual reduction in the intensity of azide stretching, highlighted in green color. The minor peak is due to the terminal azide present in the polymer chains. (C) PXRD patterns that confirm the crystalline nature of the monomer and polymeric product. The percentage of conversion mentioned in (A) refers only to the percentage of soluble oligomers in each sample, and therefore, the conversion values stated are an estimate of the reaction progression of **M** to **P**.

We observed that crystals of **M** kept at room temperature for 15 days showed decreased solubility in DMSO-*d*_6_ compared to fresh crystals, suggesting a chemical reaction of the monomer at room temperature (r.t.). For a systematic study of this spontaneous reaction, we kept 70 mg of crystals of **M** at r.t. and monitored the progress of the reaction by periodic sampling and analysis. ^1^H NMR spectra were recorded after dissolution in DMSO-*d*_6_. On the fifth day, we observed the emergence of a new signal at 7.96 ppm, attributable to the triazolyl hydrogen, H_b_, suggesting the onset of the reaction (Figure 3A).

Time-dependent ^1^H NMR analysis revealed a gradual decrease in the intensity of the alkynyl proton signal at 3.16 ppm of H_a_ and the concomitant growth of the triazolyl proton signal (Figure 3A), suggesting the progress of the reaction. After 15 days, the sample became partially insoluble. Over time, the solubility was reduced further, and from the 20th day onward, most of the sample was insoluble. As the polymer is poorly soluble in DMSO-*d*_6_, the reaction percentage calculated from the relative intensities of alkynyl and triazolyl proton signals is lower than the actual percentage. Also, the presence of only one triazolyl proton signal in the ^1^H NMR spectrum suggests regiospecific polymerization of the monomer **M** at r.t. (Figure 3A). Time-dependent IR spectroscopy study revealed gradual decrease in the intensity of the azide stretching peak at 2115 cm^–1^ (Figure 3B), and by the 30th day, the intensity of this signal reached a steady intensity, suggesting completion of the reaction. Time-dependent powder X-ray diffraction (PXRD) revealed sharp diffraction peaks continuously, evidencing retention of crystallinity during and after the reaction, although the diffraction pattern at the end of the reaction was expectedly different from the initial one (Figure 3C). Time-dependent DSC thermograms also confirmed the progress of polymerization reaction by the gradual disappearance of the exotherm at >90 °C (Figure S2). After the reaction was completed, the single crystal of the polymer retained its birefringence, as observed under a polarizing microscope (Figure 2C). We determined the structure of the product by SCXRD analysis of a single crystal that has been aged at r.t. for 30 days. The monomer **M** spontaneously evolved into a polymer in a single-crystal-to-single-crystal (SCSC) fashion. It is to be noted that there are only a few reported cases of spontaneous SCSC polymerizations.^28^ The space group (*P*1) was preserved after polymerization, and the unit cell of the product contains only one repeating unit of the polymer. The unit cell parameters changed slightly; while the parameters *a* and *c* increased by 0.87 and 1.61%, respectively, the axis *b* decreased by 1.94% which causes the crystal shape to change slightly after polymerization (Figure 5B, Movie S15). As anticipated from the molecular packing of the monomer, the topochemical polymerization occurred along the crystallographic *c* axis, resulting in the regiospecific formation of the 1,4-triazolyl-linked polymer. The triazole unit and its substituents are disordered over two positions with an occupancy ratio of 0.55:0.45 (Figure S5). The conformation of the repeating unit is similar to that of the monomer; the CH_2_–triazole and amide side chains are oriented in mutually opposite directions from the squaramide plane. This conformation is stabilized by two intramolecular CH···O hydrogen bonds (Figure 2D). The plane of the triazole ring, in both disordered forms, is almost parallel to the plane of the squaramide ring. Similar to the crystal of the monomer, the squaramide units of adjacent chains are hydrogen-bonded along the *b* axis in a β-sheet-like arrangement (Figure 2D). The NH···O hydrogen bonds between the amide units, the CH···O hydrogen bonds, and two bifurcated CH···π interactions involving carbonyl−π interaction^29^ per repeating unit connect the polymer chains along the *a* axis.

### Rapid Single-Crystal-to-Single-Crystal Polymerization at 60 °C

In order to speed up the reaction, we heated the crystals of the monomer at 60 °C and monitored the reaction progress by using IR spectroscopy. We found that at that temperature, the reaction was completed in 24 h, as shown by the negligible intensity of the azide stretch at 2115 cm^–1^ (Figure S4), and the crystal size slightly shrank (Figure 5B, cyan highlight). SCXRD analysis revealed that the crystal structure was similar to that of the polymer formed spontaneously at r.t. (Figure S6, Table S3).

### Thermoresponsive Crystal Behavior

The macroscopic changes during SCSC transformations can often be visualized by polarizing microscopy.^30−34^ To monitor the changes in the crystal, we set out to observe the crystal by using a microscope while heating it at different heating rates (1 to 20 °C/min and 13 °C/s) (Movies S1–S14). When crystals of **M** were heated at 1 °C/min, there were no observable changes (Movie S3). At a heating rate above 2 °C/min, however, the crystals showed sudden and visible cracking (Movie S1). To our surprise, the cracks closed immediately after they formed, in an impressive demonstration of self-healing behavior. This cracking and healing occur in a small temperature window of 84–90 °C, irrespective of the heating rate. As the heating rate was increased, however, the cracks formed became wider and longer, while at lower heating rates, they were smaller. At 2 °C/min, cracks of length 104 μm and width 10 μm were observed in a typical sample crystal (Movie S1). However, when heated at 20 °C/min, the cracks became more prominent, and a representative crystal showed a crack that was 265 μm long and 24 μm wide (Movie S2). It is to be noted that while the majority of the cracks healed completely, a few larger cracks remained unhealed. A crack-healed crystal, when fully reacted, was analyzed by SCXRD and had a similar structure to that of the polymer obtained after spontaneous polymerization (Table S3, Figure S7).

To obtain further insights into the origin of cracking and recovery, we recorded a DSC thermogram of monomer **M** (Figure 5C). Interestingly, we observed a sharp endothermic peak around 83–84 °C, which is followed by a much larger exothermic peak. The exothermic peak is attributed to the heat released during the azide–alkyne cycloaddition reaction, while the endothermic peak indicates a phase transition of **M** to a new polymorphic phase **M′**. We conducted DSC experiments at different heating rates ranging from 20 °C/min to 1 °C/min (Figure 5D). With an increase in heating rates, the exothermic peak is broadened and shifted toward higher temperatures as expected,^35^ however the enthalpy changes are more or less similar irrespective of heating rates (Table S1, Figure S3). As the heating rate was decreased, the endothermic peak diminished; finally, at 1 °C/min, the endothermic peak could not be observed in the thermogram. This is in agreement with the cracking behavior exhibited by the crystals, which tended to crack and heal at higher heating rates (>2 °C/min) and remained unaffected at lower heating rates. Furthermore, the perfect alignment of the cracking temperature with the endothermic thermal event strongly suggests that the cracking is due to this phase transition.

To confirm if the cracking was caused by the phase transition at 83–84 °C, a sample of **M** was rapidly heated at 13 °C/s from 50° to 85 °C, held at 85 °C for 3 s, and then cooled to 50 °C at 13 °C/s using a VAHEAT heat stage (Figure 5A). This rapid heating and cooling cycle of 50° to 85° to 50 °C was repeated three times (Movie S14). The flash heating and cooling of the sample are optimal for minimizing the polymerization reaction, as slow heating to 84 °C can lead to **M** to **P** polymerization in a time-dependent manner, diminishing the monomer **M** for phase change. When the sample was rapidly heated to 85 °C, the crystal noticeably cracked and partially healed when cooled to 50 °C, which confirms the cracking and healing processes occur via a phase transition (Figure 5A). We additionally confirmed the phase change was responsible for the healing using Raman spectroscopy on a crystal of **M** before and after the thermal cycling of 50 to 85 °C (Figure S8). Raman spectra of **M** before heating and after three thermal cycles of 50 to 85 °C confirm that after thermal cycling and cracking/healing, the crystal is in the **M** phase.

**Figure 4:**
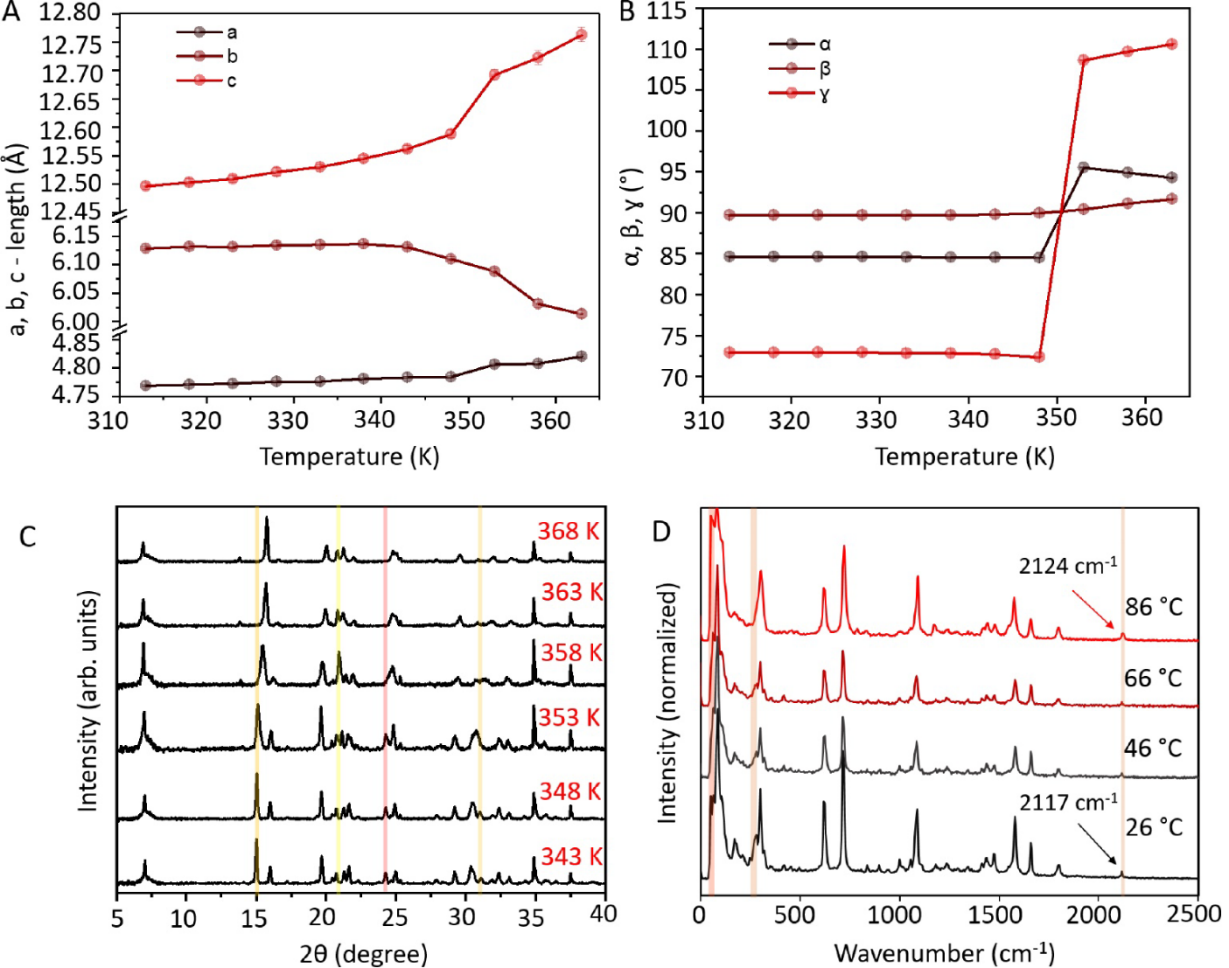
Polymorphic transition of monomer **M** to **M′**. (A) Unit cell axes as a function of temperature. (B) Unit cell angles as a function of temperature. (C) Temperature-dependent powder X-ray diffraction. (D) Temperature-dependent Raman spectra.

As the polymerization reaction competes with the phase transition, it was very difficult to trap the pure **M′** phase and determine its structure crystallographically. SCXRD analysis of a monomer crystal at 83 °C revealed a monomer with azide and alkyne groups disordered over multiple sites (CCDC 2378200), presumably due to the rapid development of a bimorphic phase (**M′** and **P**) within the crystal lattice (Figure S9). However, unit cell measurement as a function of temperature, from 313 to 363 K, (Table S2) showed a sudden and drastic change in the cell parameters at 353 K (Figure 4A,B) that did not match with that of either **M** and **P**, suggesting the transformation to a new phase, **M′**. Furthermore, temperature-dependent PXRD measurements revealed distinct diffraction patterns at 80 and 85 °C, confirming the presence of the new phase **M′** (Figures 4C and S11). Temperature-dependent Raman spectroscopy showed a shift in the signal due to alkyne from 2117 cm^–1^ (**M**) to 2124 cm^–1^ (**M′**) and appearance of shoulder peaks at 60 and 282 cm^–1^, when the sample was heated to 86 °C, further substantiating the existence of the polymorphic phase **M**′ (Figure 4D). Additionally, when the sample was heated further from 86 to 120 °C and held at 120 °C for 13 min, new peaks corresponding to phase **P** emerged at 782, 1020, 1234, and 1554 cm^–1^. As expected, the signal from the alkyne at 2124 cm^–1^ disappeared upon polymerization (Figure S10).

**Figure 5:**
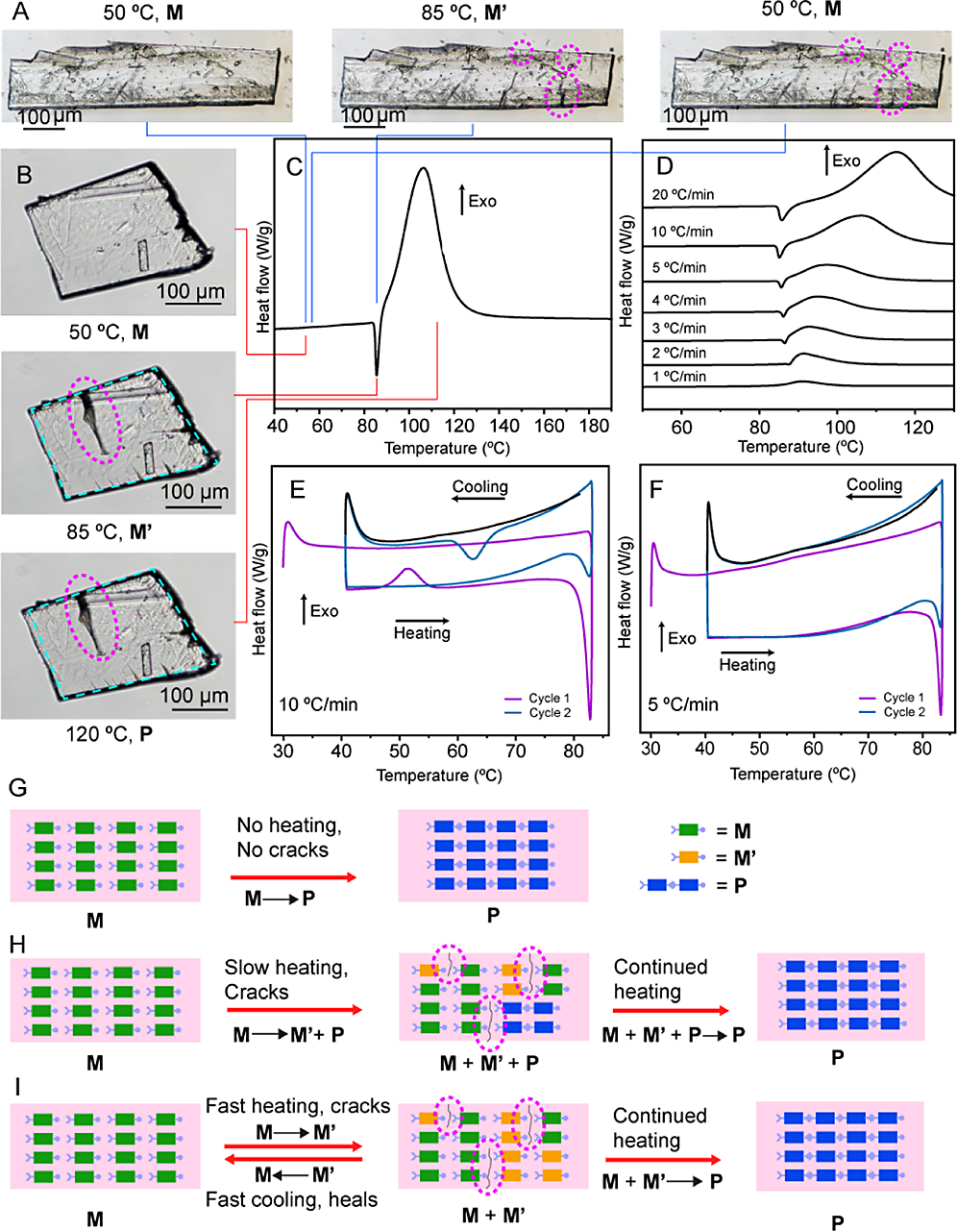
Formation and healing of cracks upon heating and cooling, and thermal profile of **M**. (A) Microscopic image of a single crystal before heating, after heating to 85 °C, and cooling to 50 °C. (B) Microscopic images of a single crystal that was heated from 50 to 120 °C and held at 120 °C for 10 min. The evolving cracks are highlighted with magenta dotted lines. (C) DSC thermogram recorded by heating the monomer **M** powder at 10 °C/min. (D) Dependence of the DSC thermograms of monomer **M** on the heating rate. (E) DSC recorded for two heating–cooling cycles of the monomer **M** at a rate of 10 °C/min. (F) DSC recorded for two heating–cooling cycle for monomer **M** at a rate of 5 °C/min. (G–I) Animated diagrams of how cracks are formed at different heating rates. (G) At room temperature, the reaction progresses from **M** to **P** without cracks. (H) At slow heating rates (<10 °C/min), cracks are formed as a result of the development of strain at the phase boundaries between **M**, **M′**, and **P**, and heal as the reaction progresses. (I) At fast heating rates (≥10 °C/min) cracks are formed by strain at the phase boundaries between **M** and **M′**, and heal as the reaction progresses to **P** upon continued heating or reverses to pure **M** upon fast cooling.

To better understand the thermal profile of **M**, we recorded the differential scanning thermogram while heating themonomer **M** up to 84 °C and subsequently cooling it back to room temperature in two consecutive cycles (Figure 5E). At a heating/cooling rate of 10 °C/min, in the first cycle, we observed an exothermic peak with an onset temperature of 55 °C during cooling, which confirmed the reversibility of the polymorphic transition. The reversibility of the polymorphic transition was visibly observed by cracking and healing when a crystal was thermally cycled from 50 to 85 °C (Movie S14). In the second cycle, a drastic reduction in the intensity of the endothermic peak on heating and the loss of reversibility on cooling were observed. When the same experiment was conducted at a heating rate of 5 °C/min for two cycles, however, the reversibility could no longer be observed (Figure 5F). It is to be noted that the monomer is reactive from r.t. onward. Two factors that determine the extent of reaction are the temperature and duration of heating; at r.t., the reaction completes in 30 days. At 60 °C the reaction completes in 24 h, while in the initial 1 h it advances to 16% completion. These collective information allow us to construct the thermal profile of crystals of **M** when it is heated under different conditions (Figure 5G–I). When a crystal of **M** is kept at r.t., it is below the phase transition temperature, and **M** slowly converts to **P** (Figure 5G). When the crystal is heated slowly, at a rate slower than 10 °C/min, the reaction of **M** to **P** occurs while it is being heated up to 84 °C; once it reaches 84 °C, **M** undergoes a polymorphic transition to **M′**. Above 84 °C, **M′** reacts to form the polymer **P** (Figure 5H). Under the slow heating conditions, there is a considerable advancement of the reaction before the phase transition, and this explains the absence of cracking or an endothermic peak at a heating rate of 1 °C/min. When a crystal is heated quickly, at a rate higher than 10 °C/min, the reaction time of **M** to **P** is minimized and **M** primarily forms **M′**. **M′** then reacts to form **P** on further heating or transforms back to **M** on sudden cooling (Figure 5I). In summary, phase **M** transforms into phase **P** when heated at a low temperature for longer period without changing to **M′** phase. However, at higher temperatures, phase **M** quickly transforms into phase **M′**, which then rapidly leads to the formation of phase **P**. We conclude that in this rare solid-state reactive system the chemical reaction competes with the phase transition.

**Figure 6:**
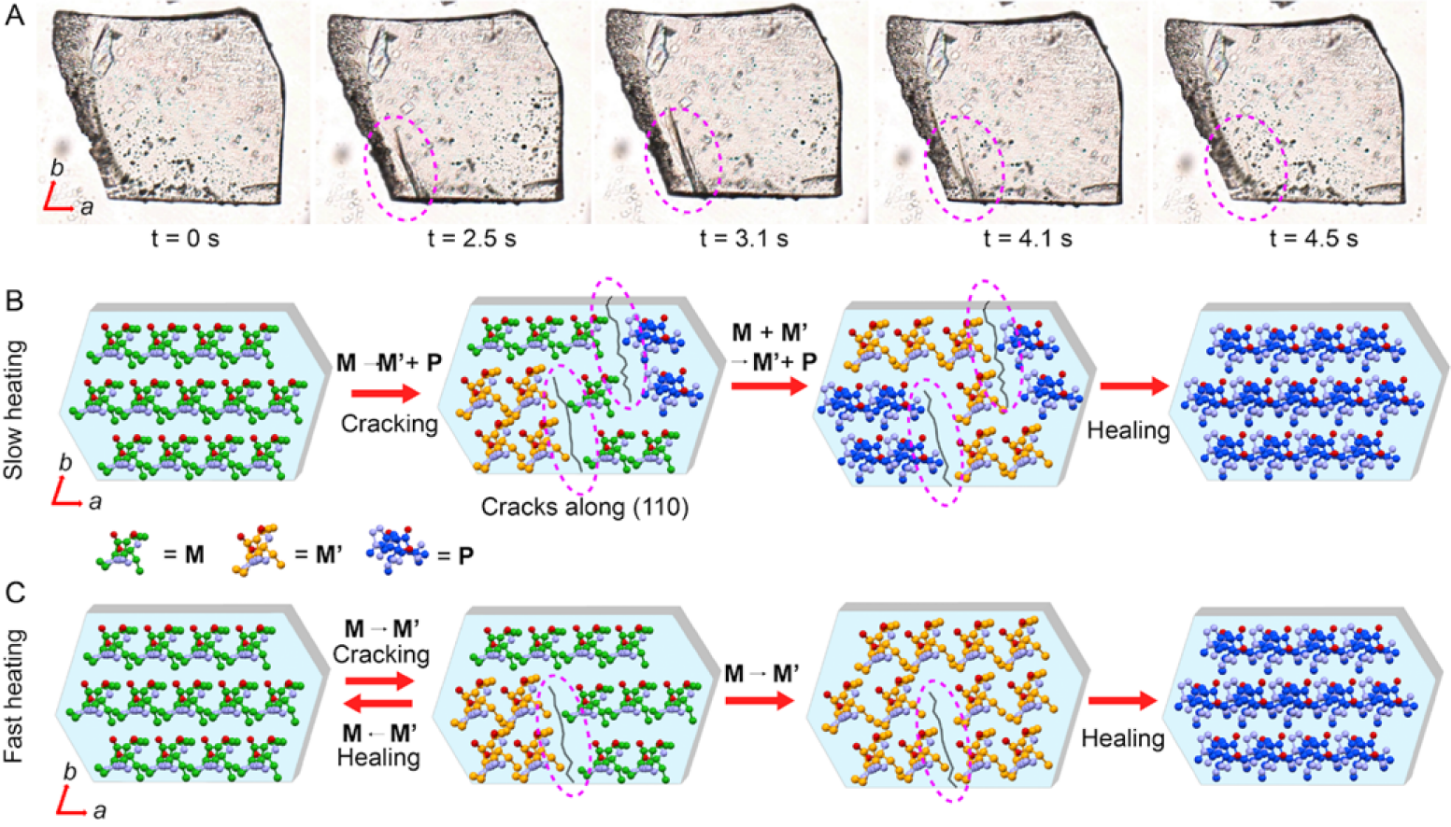
Mechanisms of self-healing and its relation to the crystal structure. (A) Microscopic images of cracked and healed crystals. (B,C) Schematic representation of two plausible pathways for the observed cracking and self-healing.

This is the reason for the reduced intensity of the endothermic peak and the loss of reversibility at a heating rate of 5 °C/min, and the similar behaviors in the second cycle, at a heating rate of 10 °C/min. To obtain further insights and understand the cracking and healing behavior, all major faces of a monomer crystal were indexed (Figure S12). When the crystal was heated on its major (001) face, it cracked primarily along the *b* axis, as shown in Figure 6A and Movie S4. At *t* = 0 s, the crystal is pristine but at 2.5 s, a crack is formed along the *b* axis that elongates at the 3.1 s mark, and then heals after 4.5 s. We have analyzed the modeled (BFDH) morphology and the energy frameworks to correlate the relationship between the cracking and the molecular interactions present in the crystal (Figures S13 and S14). The total interaction energy between the adjacent molecules arranged along the *b* axis is −81.2 kJ/mol and along the *a* axis it is −50.5 kJ/mol. This anisotropy is in agreement with the noncovalent interactions in these directions (Table S4). The relatively weaker interactions along the *a* axis allow the crystal to crack by breaking the interaction in this direction, and in effect they turn the crystal prone to cracking parallel to the *b* axis.

Analysis of the crystal structures of the monomer and polymer gives further insights into the mechanism. The N···O distance between the carbonyl oxygen and nitrogen atoms (N2···O2 and N3···O3) of adjacent squaramide units in the monomer is 2.9 Å and the angles C7–N2···O2 and C10–N3···O3 are 118.4° and 154.8° respectively. However, in the polymer, the N···O distance has been reduced to 2.8 Å, and the angles C7–N2···O2 and C10–N3···O3 have changed to 127.7° and 142.0°, respectively (Figure S15). Clearly, before the polymerization reaction, the squaramide units in **M** have slid and moved closer and presumably formed the polymorphic **M′** state. This would affect the H-bonding and other noncovalent interactions along the *a* axis in the bimorphic state where both the monomer **M** and the polymorph **M′** coexist. The strain generated in this bimorphic crystal could result in splitting across the *a* axis. At room temperature, the polymerization happens spontaneously but slowly, and the strain accumulated between the **M** and **P** domains dissipates through molecular vibrations and slight molecular reorganization in the crystal lattice. In contrast, at slow heating rates, the phase transition competes with the polymerization, resulting in the formation of multiple domains (the monomers **M** and **M′**, and oligomers/polymers), leading to the development of a significant strain between the **M**, **M′**, and **P** domains which causes cracks (Figure 6B). As the reaction progresses, the strain between different domains is removed as the samples react to yield more uniform **P** domains. At fast heating rates, lattice strain develops and cracking occurs between the **M** and **M′** domains; once a uniform **M** phase (on fast cooling) or **P** phase (on further heating or holding) is formed, the strain is removed and the cracks are healed (Figure 6C).

## Conclusion

Crystal self-healing is a rare and intriguing phenomenon that currently attracts much interest in view of its potential applications. Understanding the healing perfection requires techniques, such as X-ray crystallography; a perfectly healed crystal should be as good as a pristine crystal and should be able to determine its structure by single-crystal X-ray crystallography. We investigated crystal cracking and self-healing as a function of the heating rate during a topochemical polymerization reaction. A designed monomer undergoes SCSC polymerization via topochemical click reaction, slowly and spontaneously at r.t., but it transforms to another polymorph quickly and polymerizes upon heating. These crystals show heating-rate-dependent cracking; as the heating rate is increased, the cracks developed are larger and wider. The cracked crystals heal either upon sudden cooling due to reverse phase transition or upon further heating as a result of polymerization. The self-healed crystals retain their single-crystalline nature, which allows structure determination of a self-healed crystal by SCXRD. It is apparent that the sudden release of the strain accumulated in the crystal in view of the phase transition results in cracking and the attractive noncovalent forces between cracked surfaces help in the self-healing at atomic resolution. Such self-healing crystals might have interesting applications as smart materials.

## Supporting Information Available

Materials and methods, ^1^H NMR, ^13^C NMR, DEPT, COSY, NOESY, HMBC, and HMQC spectra for the monomer; additional experimental details, including PXRD analysis, Raman spectra, energy framework analysis; interaction table; and crystallographic table (PDF)
The macroscopic changes of **M** during SCSC transformation at 2 °C/min (MP4)
The macroscopic changes of **M** during SCSC transformation at 20 °C/min (MP4)
The macroscopic changes of **M** during SCSC transformation at 1 °C/min (MP4)
The macroscopic changes of **M** during SCSC transformation at 3 °C/min (MP4)
The macroscopic changes of **M** during SCSC transformation at 4 °C/min (MP4)
The macroscopic changes of **M** during SCSC transformation at 5 °C/min (MP4)
The macroscopic changes of **M** during SCSC transformation at 6 °C/min (MP4)
The macroscopic changes of **M** during SCSC transformation at 7 °C/min (MP4)
The macroscopic changes of **M** during SCSC transformation at 8 °C/min (MP4)
The macroscopic changes of **M** during SCSC transformation at 9 °C/min (MP4)
The macroscopic changes of **M** during SCSC transformation at 10 °C/min (MP4)
The macroscopic changes of **M** during SCSC transformation at 12 °C/min (MP4)
The macroscopic changes of **M** during SCSC transformation at 16 °C/min (MP4)
The macrocopic changes of **M** during rapid heating and cooling cycle from 50 °C to 85 to 50 °C repeated three times (MP4)
The macroscopic changes of **M** during heating from 90 °C to 120 °C over 10 minutes (MP4)
