# Supplementary material for: A Self-Healing Crystal That Repairs Multiple Cracks
Source: J Am Chem Soc. 2024 Sep 18;146(39):27100–8. doi: 10.1021/jacs.4c09334 (PMC11457417; doi:10.1021/jacs.4c09334)
Supplement: Supplementary file 1 — ja4c09334_si_001.pdf [file ja4c09334_si_001.pdf]

Supplementary Materials for

A Self-Healing Crystal that Repairs Multiple Cracks

Javed R. Pathan, Haripriya Balan, Patrick Commins, Arthi Ravi, Marieh B. Al-Handawi, Ian Cheng-Yi Hou, Pančе Naumov* and Kana M. Sureshan*

*Email: kms@iisertvm.ac.in, pance.naumov@nyu.edu

Table of content

Sl. No.	Section	Page No
1	Materials and methods	2
2	Synthesis scheme	3
3	PXRD comparison	4
4	Time-dependent DSC analysis	5
5	Heat effects of the reactions	6
6	FT-IR spectra of monomer and resulting polymer	7
7	ORTEP diagrams	8
8	Crystal structure comparison of polymer obtained at rt and 60 ^oC	8
9	Crystal structure comparison of polymer obtained at rt and after healing	9
10	Raman spectra after three thermal cycles	9
11	Overlay image of monomer **M** and **M'**	10
12	Temperature dependent Raman spectra	10
13	Temperature dependent PXRD	11
14	Face indexing of **M**	11
15	Modelled morphology and energy framework	12
16	Energy framework calculations	12
17	Analysis of crystal structure of **P**	13
18	Table S2. Changes in the unit cell parameters with temperature	13
19	Table S3. Crystallographic information of the monomer and the polymer obtained under different conditions	13
20	Table S4. Interaction table for the monomer and the polymer	14
21	Spectral information	16
22	References	19
23	Movie captions	19

1. Materials and methods

All required chemicals, such as L-alanine, dimethyl squarate, di-tert-butyl dicarbonate, propargylamine, 2-aminoethanol, triethylamine, and calcium hydride were purchased from Spectrochem and used as such without further purification. The TLC plates were purchased from Merck.

All synthesized intermediates and final monomer derivatives were purified using thin-layer chromatography using 200-400 mesh silica gel. The chromatograms were visualized after dipping in a ceric ammonium molybdate solution, followed by charring by hot air gun. Avance III-500 (Bruker) NMR spectrometer was used to record the ^{1}H NMR, ^{13}C NMR, DEPT, COSY, NOESY, HMBC, and HMQC. ^{1}H NMR spectra were recorded at 500 MHz, and the ^{13}C NMR spectra were recorded at 125 MHz after dissolving the compound in deuterated chloroform ($CDCl_3$) or dimethylsulfoxide (DMSO-d_6). The chemical shifts of the proton (δ) and carbon signals are reported in ppm. Tetramethylsilane was used as the reference (TMS, δ = 0.0). The coupling constants (*J*) were described in Hertz, and spin multiplicities were denoted as singlet (s), doublet (d), triplet (t), and multiplet (m) etc. IR spectra were recorded on an IR Prestige-21 instrument after making thin pellets of the compound with KBr. Melting points were recorded on a Stuart SMP 30 melting point apparatus. Differential Scanning Calorimetry (DSC) thermograms were recorded on DSC Q20 Differential Scanning Calorimeter. Flash heating experiments at 13 ºC/s were performed on a VAHEAT SmS-E model microscope heating stage. Images of the flashing heating experiments were captured on a Nikon-LV Dia microscope using a Plan Fluor 10x objective. The Raman spectra of 2 were collected by using a Witec Alpha 300 confocal Raman spectrometer under a 50x objective with a numerical aperture (N.A.) of 0.8. A 785 nm laser was used with a total acquisition time of 1 s. Thermogravimetric analysis (TGA) was carried out on a Universal V4.7A TA instrument. Powder X-ray diffraction analyses were carried out on a Bruker D8 ADVANCE powder X-ray diffractometer with a Cu source (CuKα = 1.5418 Å). Single crystal X-ray intensity data were collected on a Bruker AXS Kappa APEX II diffractometer using Mo Kα radiation (λ = 0.710 73 Å). Refinement was carried out with Olex2-1.5. Optical microscope images were recorded with a Qimaging Qclick camera mounted on the Olympus BX51 microscope.

2. Synthesis scheme

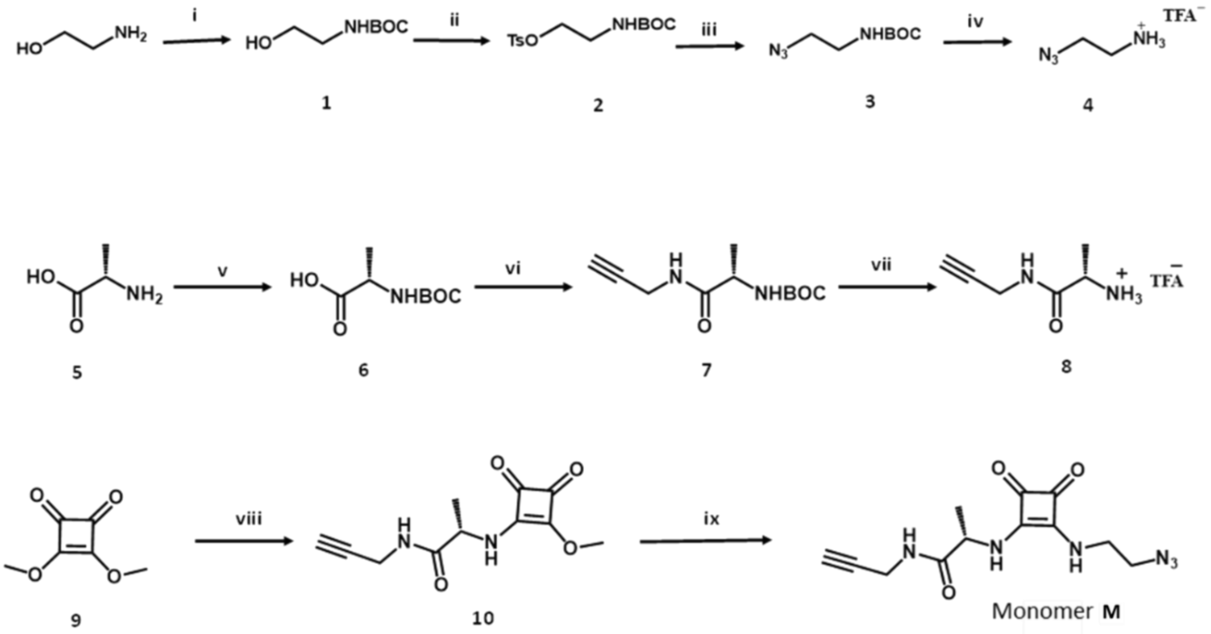

Scheme 1: Synthesis of monomer **M** i) Boc anhydride, Et_3N, DCM, 75% ii) Tosyl chloride, Et_3N, DCM, 60% iii) NaN_3, DMF, 60 ºC, 80% iv) TFA, DCM. v) Boc anhydride, NaOH, THF: water, 68% vi) Propargylamine, EDC.HCl, HOBt, Et_3N, DCM, 76 % vii) TFA, DCM. viii) **8**, Et_3N, methanol, 84% ix) **4**, Et_3N, methanol, 81%.

Synthesis of compound **4:**
The azidoamine was synthesized as reported previously [1].

Synthesis of compound **6:**
To a solution of L-alanine **5** (2g, 22.45 mmol) in 1:1 mixture of dioxane and water (80 mL), aqueous solution of NaOH (1.79 g, 44.91 mmol in 10 mL of water) was added. Later, Boc anhydride (5.67 mL, 24.70 mmol) was added dropwise and the reaction mixture was stirred for another 5 h at room temperature. After complete consumption of the starting material as monitored by TLC, solvent was removed under reduced pressure. The residue thus obtained was acidified using dilute HCl solution and extracted using ethyl acetate thrice. Organic layer was dried over anhydrous Na_2SO_4. The solvent was evaporated to obtain compound **6** (2.9 g, 68 %) as an oil. The crude compound was used in the next step [2].

Synthesis of compound **7**:
To a solution of compound **6** (2 g, 10.57 mmol) in dry DCM (50 mL), EDC.HCl (2.2 g, 11.63 mmol) and HOBt (1.57g, 11.63 mmol) were added portion wise at 0 ºC, and the solution was kept for stirring at the same temperature for 50 min. Later, propargylamine (0.744 mL, 11.63 mmol) and triethylamine (4.4 mL, 31.71 mmol) were added dropwise to the above reaction mixture and allowed to stirred at room temperature for overnight. The solvent was removed under reduced pressure and the residue was extracted with ethyl acetate, washed thrice with saturated sodium bicarbonate and brine. The organic layer was dried over anhydrous sodium sulphate, evaporated under reduced pressure and the residue thus obtained was purified using column chromatography, using 1:1 mixture of ethyl acetate and petroleum ether as eluent, to obtain intermediate **7** as a white solid (1.81 g, 76 %) [3].

Synthesis of compound **8:**

To a cooled (0 °C) solution of **7** (286 mg, 1.26 mmol) in DCM (20 mL), TFA (0.97 mL, 12.66 mmol) was added dropwise. Then the reaction mixture was stirred at room temperature for another 3 h. The excess TFA and DCM were evaporated under reduced pressure. The oily residue thus obtained was co-evaporated with toluene thrice and used in the next step without further purification.

Synthesis of compound **10:**
To a solution of dimethyl squarate (150 mg, 1.05 mmol) in methanol (10 mL), intermediate **8** (0.28 g, 1.26 mmol) and triethylamine (0.735 mL, 5.27 mmol) were added. After addition, the reaction mixture was kept for stirring at room temperature for overnight. After complete consumption of the starting material, as judged by TLC, the solvent was evaporated and the residue thus obtained was purified using column chromatography, using 6:1 mixture of ethyl acetate and petroleum ether as eluent, to get the compound **10** (0.21 g, 84%) as a white solid.

Synthesis of monomer **M**:
To a solution of compound **10** (200 mg, 0.84 mmol) and intermediate **4** (0.18 g, 1.01 mmol) in 10 mL methanol, triethylamine (0.59 mL, 4.23 mmol) was added and the reaction mixture was stirred at room temperature for 24h. After complete consumption of the starting material, as judged by TLC, the solvent was evaporated and the residue thus obtained was purified using column chromatography, using 8:2 mixture of ethyl acetate and petroleum ether as eluent, to yield the monomer **1** (0.2 g, 81%) as a white solid.

3. PXRD comparison

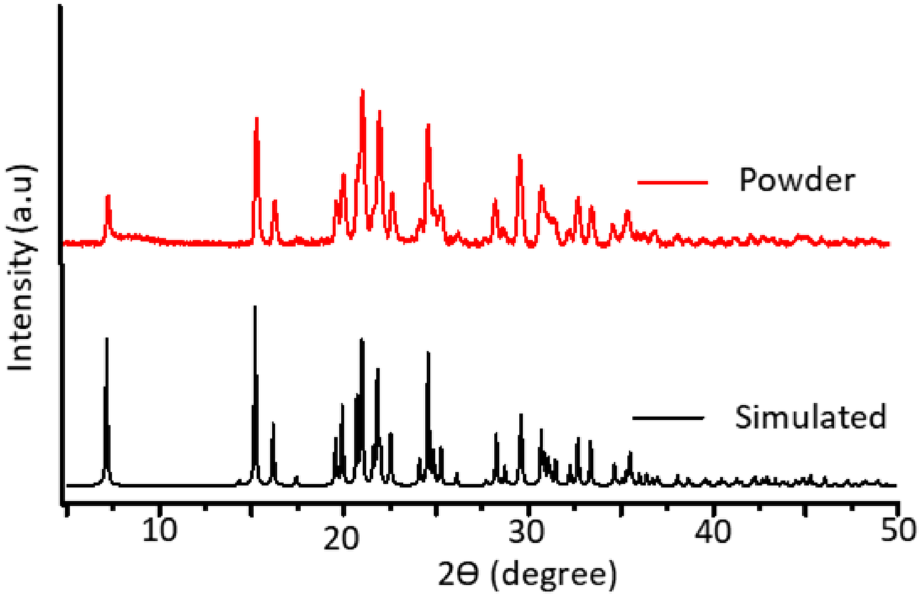

Figure S1. Overlay of experimental PXRD profile of the bulk monomer (powder) and the simulated PXRD profile generated from the crystal structure.

4. Time-dependent DSC analysis

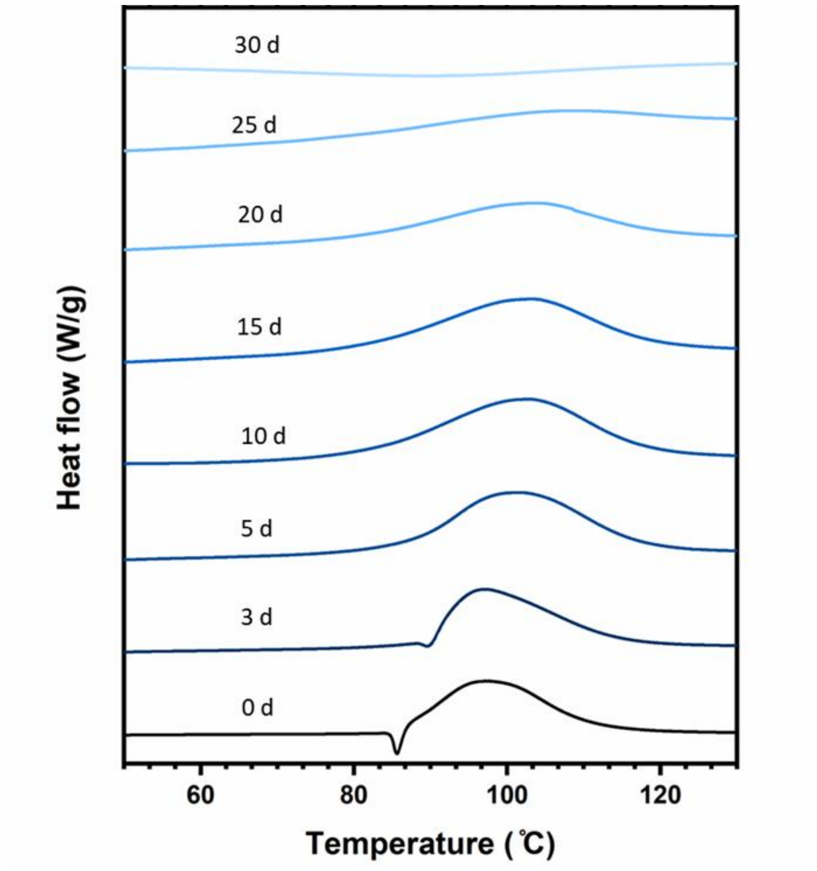

Figure S2. Time-dependent DSC thermograms during the spontaneous polymerization.

5. Heat effects of the reactions

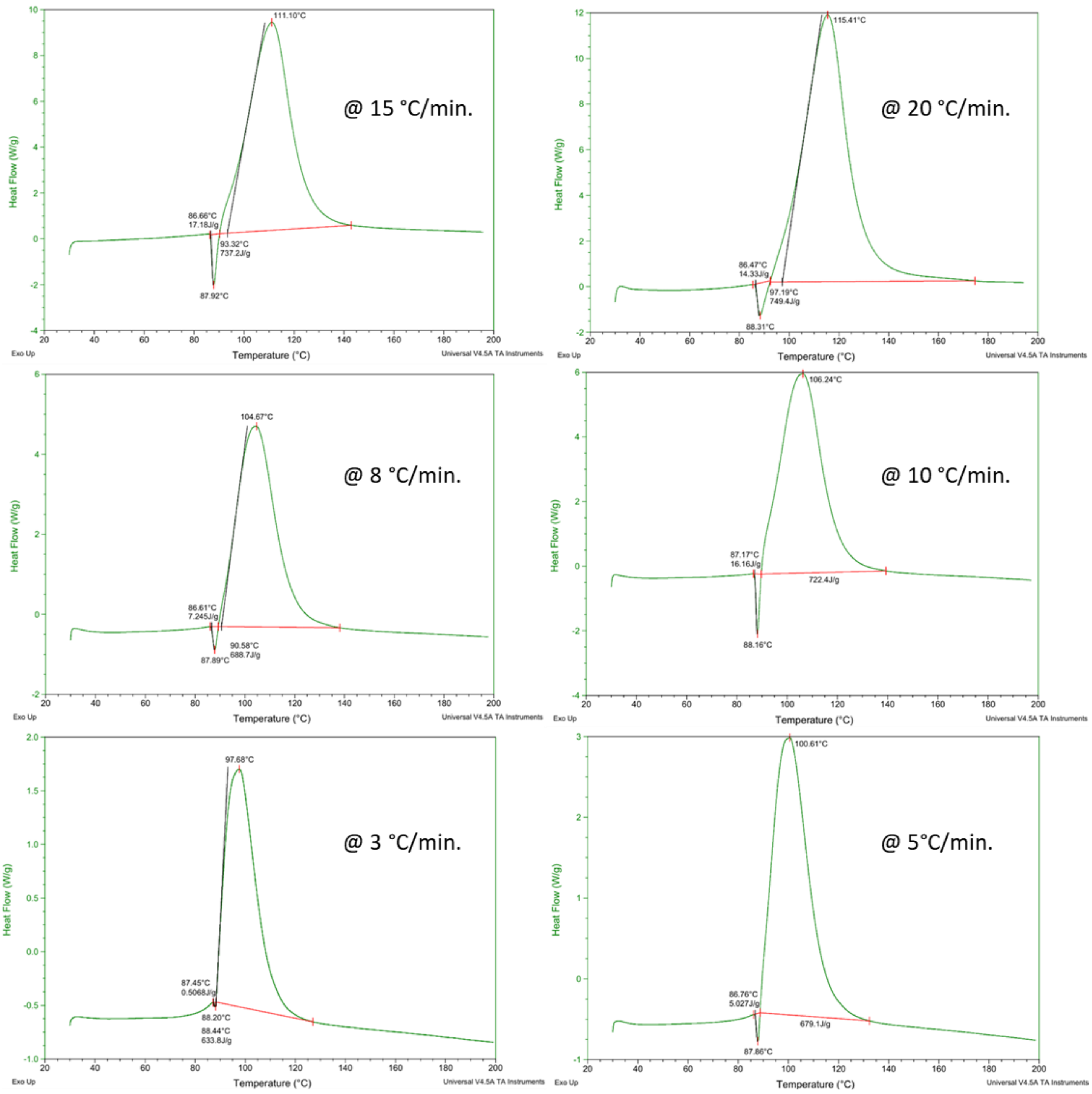

Figure S3. DSC thermograms at different heating rates.

Table S1. Heat effects of the reactions at different heating rates.

	ΔH_1	ΔH_2
@ 3 °C/min.	0.50 J/g	638.8 J/g
@ 5 °C/min.	5.02 J/g	679.1 J/g
@ 8 °C/min.	7.24 J/g	688.7 J/g
@ 10 °C/min.	16.16 J/g	722.4 J/g
@ 15 °C/min.	17.18 J/g	737.2 J/g
@ 20 °C/min.	14.33 J/g	749.4 J/g

6. FT-IR spectra of monomer and resulting polymer

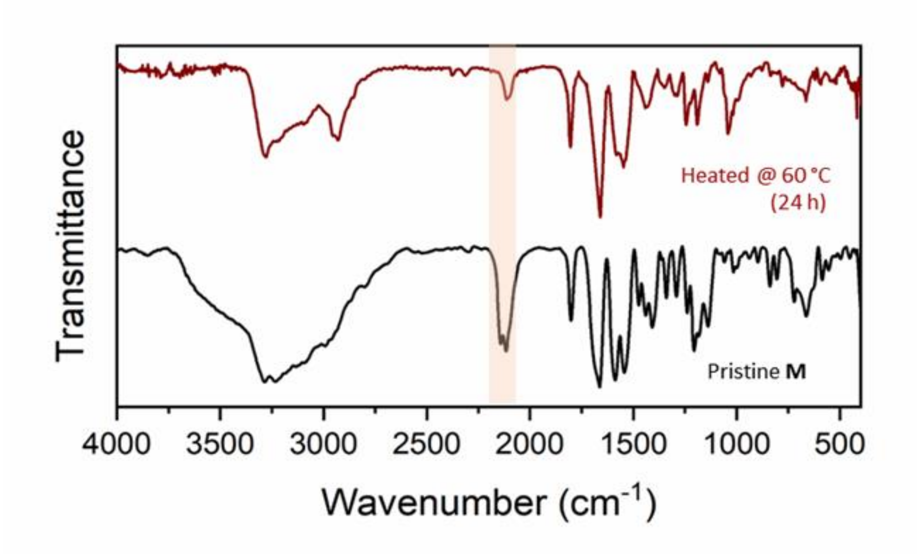

Figure S4. FT-IR analysis before and after polymerization (monomer **M** was heated at 60 °C for 24h).

7. ORTEP diagrams

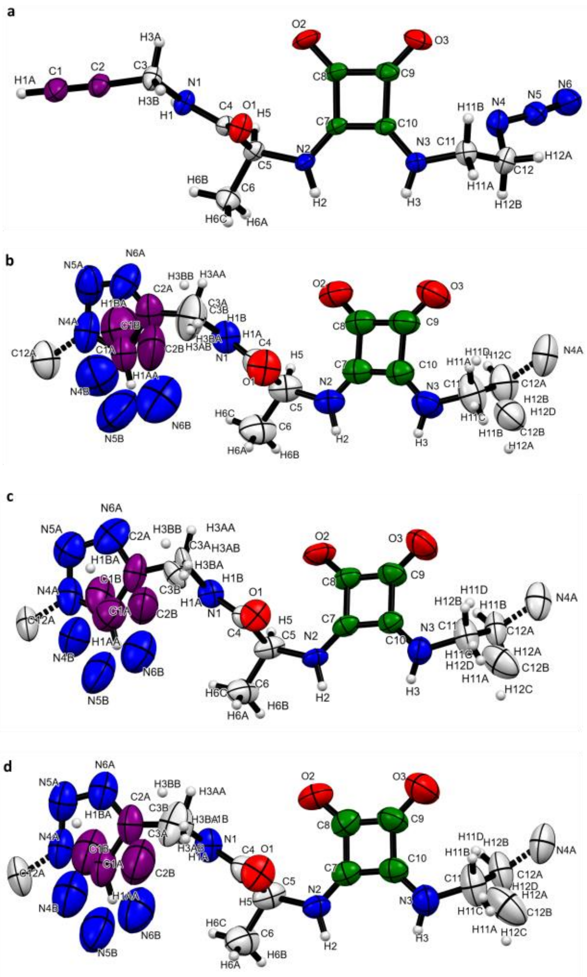

Figure S5. ORTP diagram of **a**, The monomer crystal. **b-d**, the polymer crystals formed at rt, 60 °C and after healing respectively, (Thermal ellipsoids are set at 50 % probability).

8. Crystal structure comparison of polymer obtained at rt and 60 °C

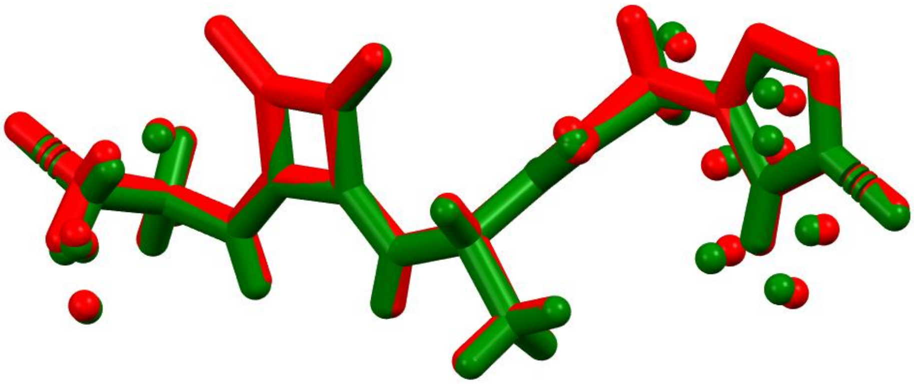

Figure S6. Overlay of crystal structures of the polymer, **P**, formed at rt (red) and at 60 °C (green).

9. Crystal structure comparison of polymer obtained at rt and after healing

As healed crystals (partially reacted) were not suitable for SCXRD analysis, we have continued the heating for a few more minutes (15 minutes) after the cracking and healing to ensure complete reaction and submitted for SCXRD analysis.

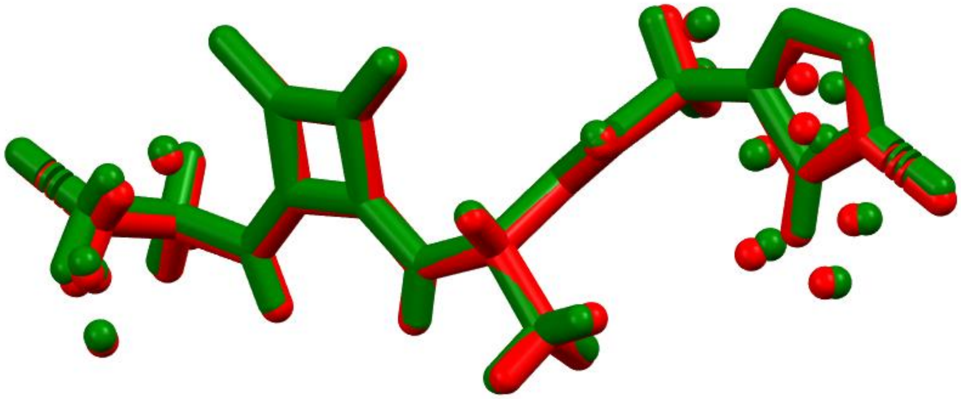

Figure S7. Overlay of crystal structures of the polymer formed at rt (red) and the one obtained after healing and complete polymerization (green).

10. Raman spectra after three thermal cycles

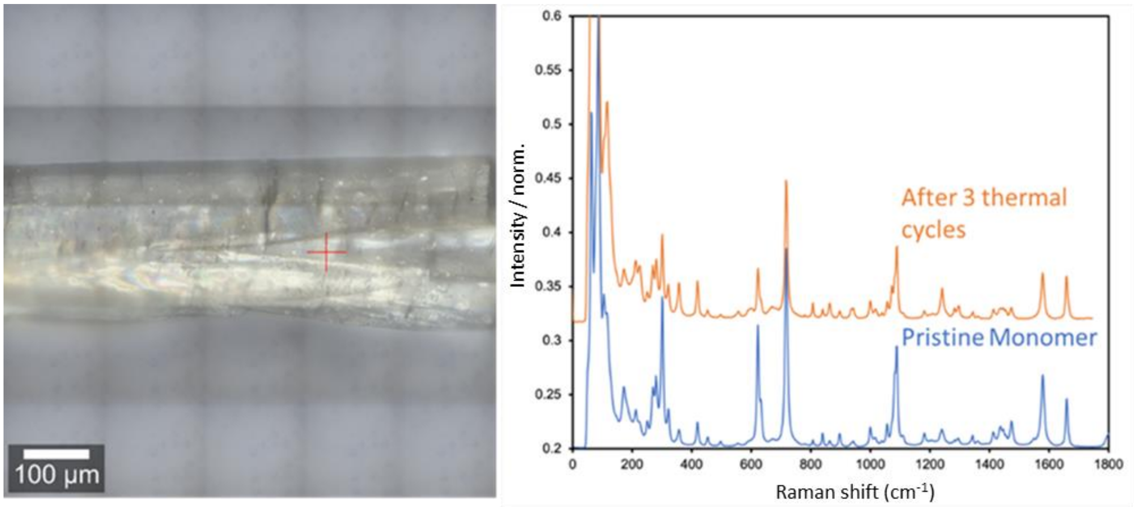

Figure S8. Raman spectra of pristine monomer and after three consecutive thermal cycles of heating from 50 to 85 ºC at 13 ºC/s.

11. Overlay image of monomer M and M’

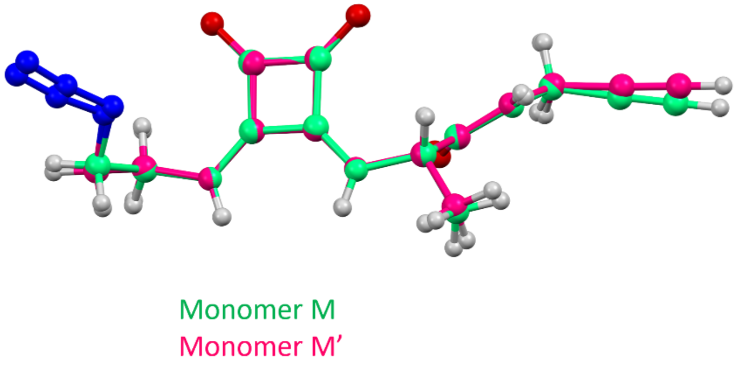

Monomer M

_cell_length_a	4.7460(12)
_cell_length_b	6.1209(16)
_cell_length_c	12.392(3)
_cell_angle_alpha	84.535(8)
_cell_angle_beta	89.462(7)
_cell_angle_gamma	72.889(7)
_cell_volume	342.42(15)

Monomer M’

_cell_length_a	4.7868(19)
_cell_length_b	6.089(3)
_cell_length_c	12.635(6)
_cell_angle_alpha	95.580(13)
_cell_angle_beta	90.075(16)
_cell_angle_gamma	108.174(14)
_cell_volume	348.0(3)

Figure S9. Overlay image and unit cell parameters of monomer **M** and **M’**.

12. Temperature-dependent Raman spectra

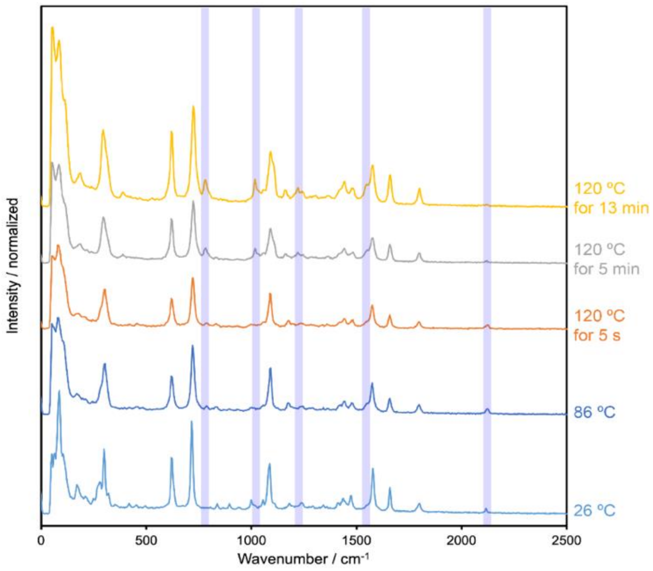

Figure S10. Raman spectra showing new peaks corresponding to phase **P** emerged at 782, 1020, 1234, and 1554 cm^{-1} and alkyne peak disappeared upon polymerization.

13. Temperature-dependent PXRD

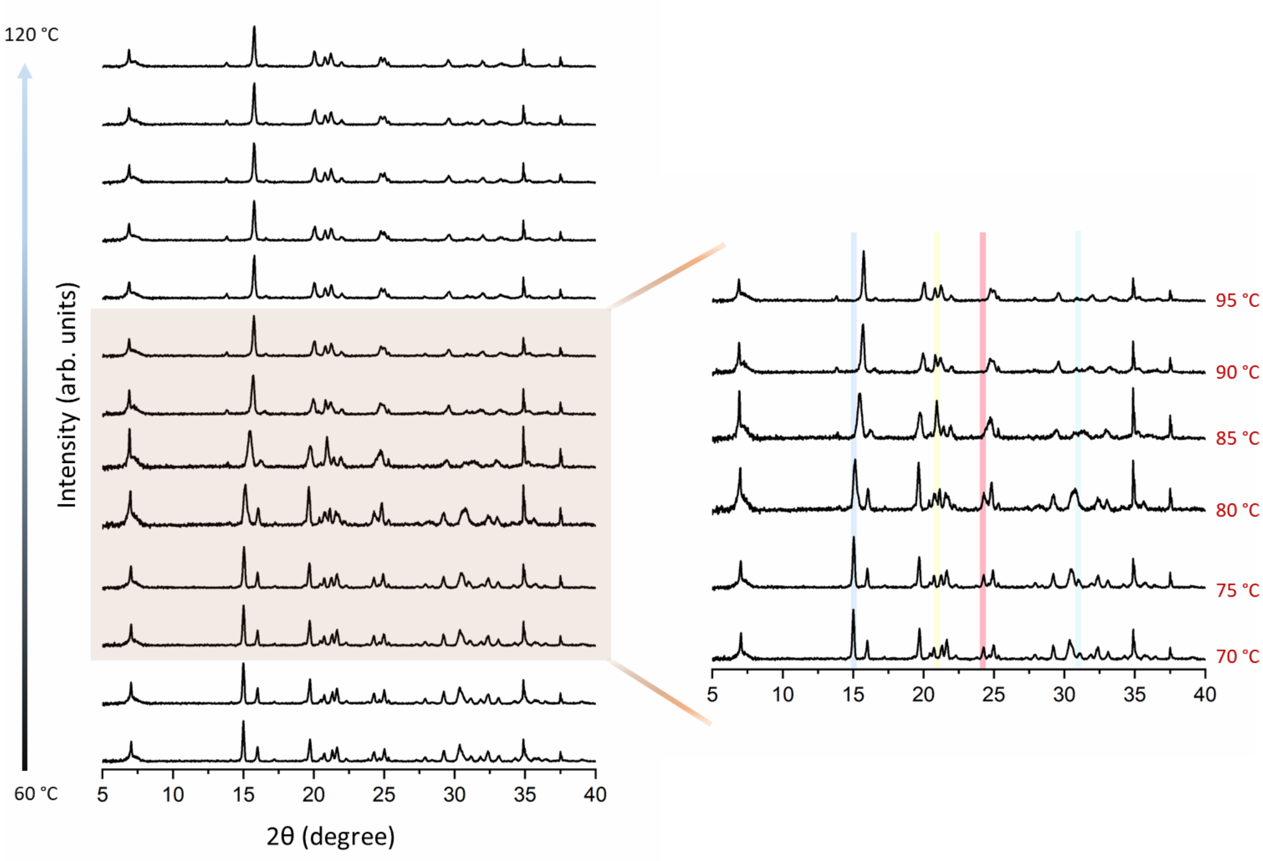

Figure S11. Temperature dependent PXRD analysis showing different diffraction pattern after 80 °C.

14. Face indexing of M

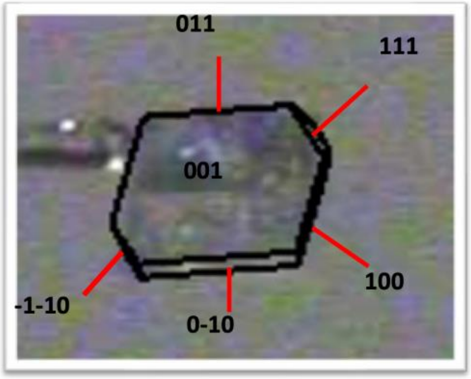

Figure S12. Face indexing for a crystal of **M.**

15. Modelled morphology and energy framework

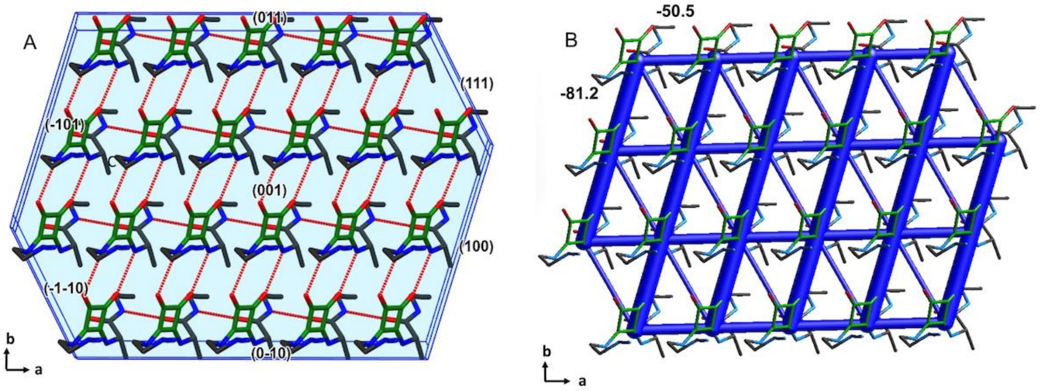

Figure S13. Modelled (BFDH) morphology (A) and energy framework (B) of **M.**

16. Energy framework calculations

Energy frameworks for monomer is constructed using the CE-B3LYP/ B3LYP/6-31G(d,p). Molecular wave functions calculated at the crystal geometry in CrystalExplorer21.5. The cylinders represent the interaction energy between the two adjacent molecules, and the radius of the cylinder corresponds to the magnitude of interaction energy. Interaction below -4 kJ/mol were omitted to avoid crowding in the framework. Total energy is decomposed into electrostatic, polarization, dispersion and exchange-repulsion components based on scaling factors 1.057, 0.740, 0.871, and 0.618 respectively.

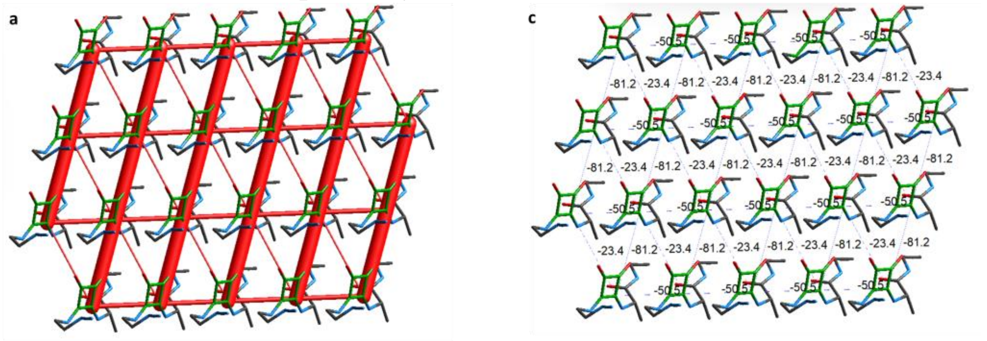

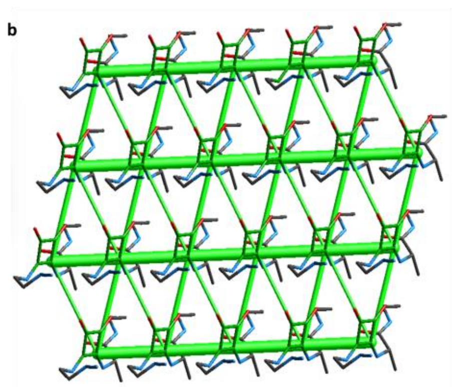

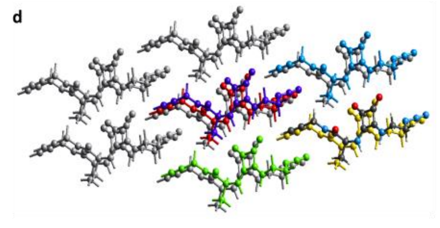

	N	Symop	R	Electron Density	E_ele	E_pol	E_dis	E_rep	E_tot
	1	x, y, z	14.67	B3LYP/6-31G(d,p)	3.0	-1.0	-7.6	6.0	-0.5
	1	x, y, z	4.75	B3LYP/6-31G(d,p)	-22.3	-19.9	-61.3	66.7	-50.5
	1	x, y, z	13.23	B3LYP/6-31G(d,p)	-18.8	-4.6	-17.6	17.3	-28.0
	0	x, y, z	6.55	B3LYP/6-31G(d,p)	-8.6	-5.5	-19.4	10.8	-23.4
	1	x, y, z	6.12	B3LYP/6-31G(d,p)	-70.2	-22.2	-31.6	60.0	-81.2
	1	x, y, z	13.29	B3LYP/6-31G(d,p)	-4.2	-1.5	-16.0	18.0	-8.4
	0	x, y, z	12.39	B3LYP/6-31G(d,p)	-2.3	-1.2	-14.7	12.6	-8.4

Figure S14. Energy frameworks corresponding to **a**, electrostatic (red), **b**, dispersion (green), **c**, molecular pairs for monomer crystal. **d**, Details of energy framework analysis of monomer crystal structure calculated using Crystal Explorer 21.5 software.

17. Analysis of crystal structure of P

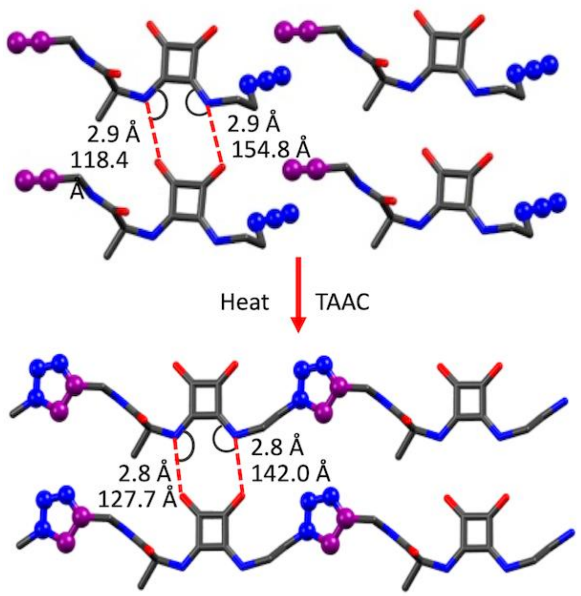

Figure S15. Analysis of crystal structure of **P** showcasing the N···O distance of 2.8 Å, and the angles C7-N2···O2 and C10-N3···O3 of 127.7º and 142.0º, which shows the squaramide units have moved closer together.

18. Table S2. Changes in the unit cell parameters with temperature

	313 K	318 K	323 K	328 K	333 K	338 K	343 K	348 K	353 K	358 K	363 K	Polymer
a (Å)	4.7687 (16)	4.7707 (16)	4.7720 (16)	4.776 (2)	4.7758 (11)	4.7808 (16)	4.7830 (17)	4.784 (2)	4.806 (3)	4.808 (3)	4.820 (4)	4.7974 (12)
b (Å)	6.129 (4)	6.132 (4)	6.131 (4)	6.134 (4)	6.1350 (19)	6.137 (3)	6.131 (3)	6.110 (3)	6.088 (4)	6.031 (7)	6.013 (7)	6.0079 (17)
c (Å)	12.497 (6)	12.503 (6)	12.509 (6)	12.521 (7)	12.530 (4)	12.545 (6)	12.562 (6)	12.588 (7)	12.692 (9)	12.723 (12)	12.763 (13)	12.708 (4)
α (°)	84.614 (16)	84.621 (17)	84.637 (16)	84.63 (2)	84.573 (9)	84.538 (12)	84.523 (13)	84.480 (14)	95.500 (16)	94.90 (3)	94.28 (3)	93.420 (9)
β (°)	89.70 (2)	89.70 (2)	89.70 (2)	89.70 (3)	89.705 (11)	89.713 (15)	89.784 (16)	89.946 (18)	90.40 (2)	91.12 (4)	91.66 (4)	92.811 (8)
γ (°)	72.910 (17)	72.906 (18)	72.941 (17)	72.92 (2)	72.832 (11)	72.826 (15)	72.689 (16)	72.325 (19)	108.64 (2)	109.70 (3)	110.59 (3)	109.848 (8)

19. Table S3. Crystallographic information of the monomer and the polymer obtained under different conditions

Identification code	Monomer **1**	Polymer **P** (at rt)	Polymer **P** (at 60 ºC)	Polymer **P** (Healed)
CCDC	2281466	2281467	2281468	2281469
Empirical formula	$C_{12}H_{14}N_6O_3$	$C_{12}H_{14}N_6O_3$	$C_{12}H_{14}N_6O_3$	$C_{12}H_{14}N_6O_3$
Formula weight	290.29	290.29	290.29	290.29

Temperature/K	100	100	100	100
Crystal system	triclinic	triclinic	triclinic	triclinic
Space group	*P*1	*P*1	*P*1	*P*1
a/Å	4.7460(12)	4.7874(10)	4.7773(13)	4.7974(12)
b/Å	6.1209(16)	6.0018(14)	5.9807(18)	6.0079(17)
c/Å	12.392(3)	12.592(3)	12.644(4)	12.708(4)
α/°	84.535(8)	93.372(9)	93.634(9)	93.420(9)
β/°	89.462(7)	93.081(8)	92.853(9)	92.811(8)
γ/°	72.889(7)	108.944(8)	109.935(9)	109.848(8)
Volume/Å^3	342.42(15)	340.59(13)	337.96(17)	342.97(16)
Z	1	1	1	1
ρ_{calc}g/cm^3	1.408	1.415	1.426	1.405
μ/mm^{-1}	0.106	0.106	0.107	0.106
F(000)	152.0	152.0	152.0	152.0
Crystal size/mm^3	0.065 × 0.045 × 0.035	0.1 × 0.05 × 0.04	0.11 × 0.069 × 0.045	0.11 × 0.085 × 0.04
Radiation	MoKα (λ = 0.71073)	MoKα (λ = 0.71073)	MoKα (λ = 0.71073)	MoKα (λ = 0.71073)
2Θ range for data collection/°	6.608 to 56.226	6.502 to 56.874	6.476 to 56.764	6.442 to 55.058
Index ranges	-6 ≤ h ≤ 6, -8 ≤ k ≤ 7, -16 ≤ l ≤ 16	-6 ≤ h ≤ 6, -8 ≤ k ≤ 7, -16 ≤ l ≤ 16	-6 ≤ h ≤ 6, -7 ≤ k ≤ 7, -16 ≤ l ≤ 16	-6 ≤ h ≤ 6, -7 ≤ k ≤ 7, -16 ≤ l ≤ 16
Reflections collected	8026	11200	9930	10677
Independent reflections	3262 [R_{int} = 0.0377, R_{sigma} = 0.0606]	3372 [R_{int} = 0.0443, R_{sigma} = 0.0679]	3341 [R_{int} = 0.0387, R_{sigma} = 0.0634]	3094 [R_{int} = 0.0422, R_{sigma} = 0.0527]
Data/restraints/parameters	3262/3/191	3372/222/255	3341/102/254	3094/125/255
Goodness-of-fit on F^2	0.989	1.125	1.014	1.015
Final R indexes [I>=2σ (I)]	R_1 = 0.0456, wR_2 = 0.0887	R_1 = 0.1120, wR_2 = 0.3031	R_1 = 0.0967, wR_2 = 0.2580	R_1 = 0.0902, wR_2 = 0.2432
Final R indexes [all data]	R_1 = 0.0770, wR_2 = 0.1010	R_1 = 0.1897, wR_2 = 0.3548	R_1 = 0.1542, wR_2 = 0.3018	R_1 = 0.1420, wR_2 = 0.2826
Largest diff. peak/hole / e Å^{-3}	0.19/-0.16	0.56/-0.31	0.46/-0.27	0.34/-0.25
Flack parameter	-1.3(9)	-0.2(10)	-0.7(9)	0.0(8)

20. Table S4. Interaction table for the monomer and the polymer

Motif	Interaction	Symmetry code	D···A (Å)/H…A(Å) /D-H…A (°)	
			Monomer	Polymer
N-H…O	N1-H1…O1	x+1,+y,+z	2.8/2.0/ 161	-
	N1-H1A-O3	x-1,+y,+z	-	2.8/1.9/171

	N2-H2…O2	x,+y-1,+z	2.9/2.0/170	-
	N3-H3-O3	x,+y-1,+z	2.9/ 2.3/ 130	-
		x,+y+1,+z	-	2.7/ 1.9/146
C-H…O	C3-H3B…O1	x,y,z	2.8/2.4/102	-
	C3A-H3AB…O3	x,y,z	-	2.8/2.4/ 99
	C6-H6A-O2	x-1,+y+1,+z	3.5/ 2.6/160	-
	C6-H6B-O2	x,+y-1,+z	-	3.3/ 2.9/102
	C5-H5…01	x+1,+y,+z	3.2/2.5/ 127	3.3/2.6/124
	C5-H5…03	x-1,+y,+z	-	3.3/2.6/124
	C5-H5…02	x,y,z	-	3.1/2.7/ 107
	C11-H11B-O3	x,y,z	3.3/2.6/127	-
	C11-H11A-O3	x,+y-1,+z	3.0/2.6/104	-
	C1-H1A-O3	x+1,+y,+z-1	3.4/ 2.5/173	-
	C12A-H12B-O3	x,y,z	-	3.3/ 2.6/121
C-H…N	C3-H3B…N6	x,+y,+z-1	3.1/2.7/106	-
	C3-H3A…N6	x,+y-1,+z+1	3.3/ 2.5/134	-

21. Spectroscopic Information

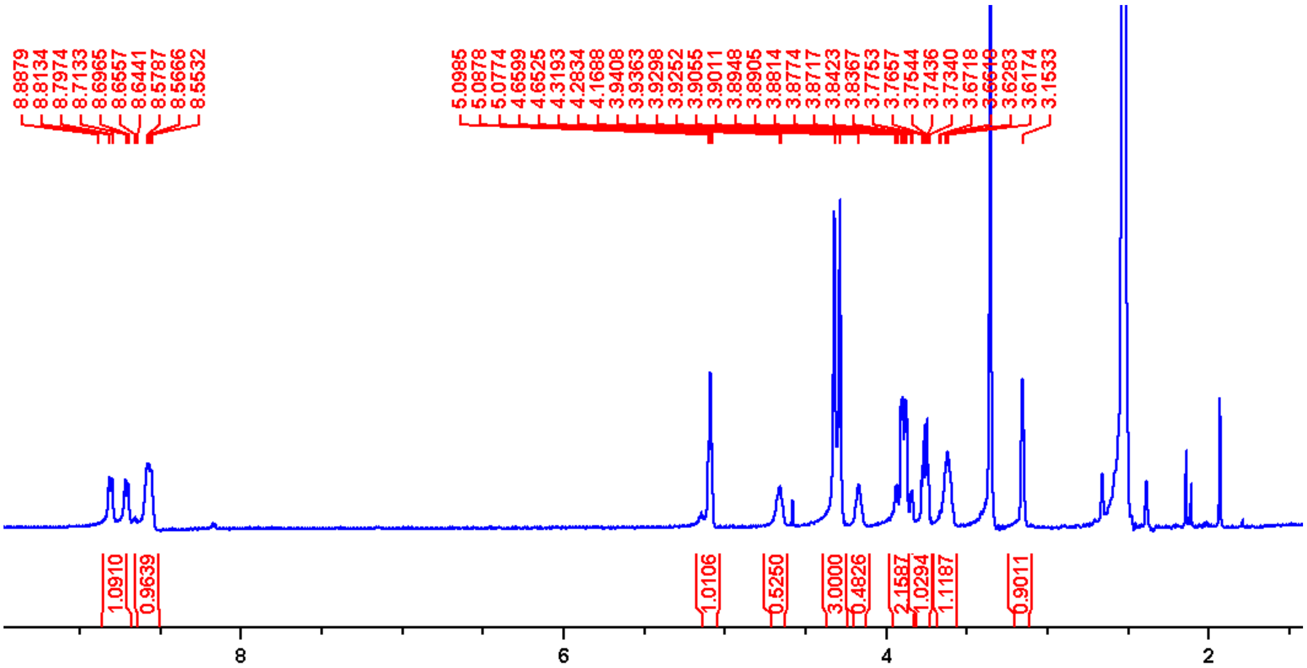

Figure S16. ^{1}H NMR spectrum of the compound **10** in DMSO-d_6

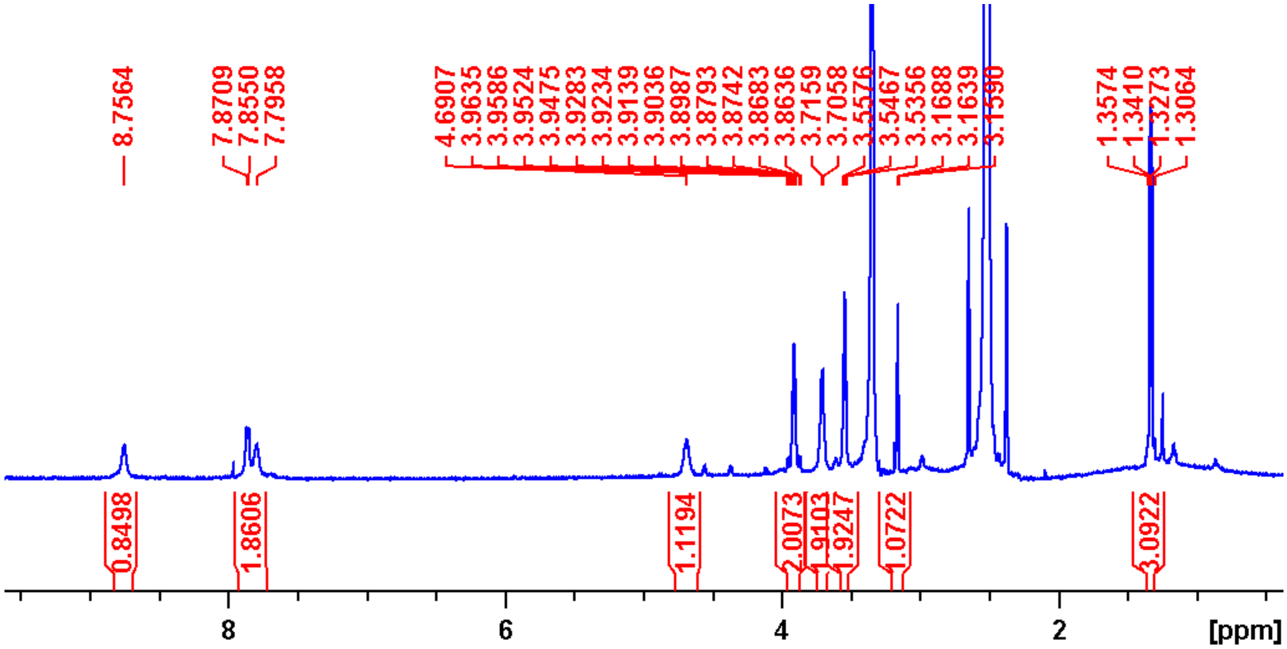

Figure S17. ^{1}H NMR spectrum of the monomer **M** in DMSO-d_6

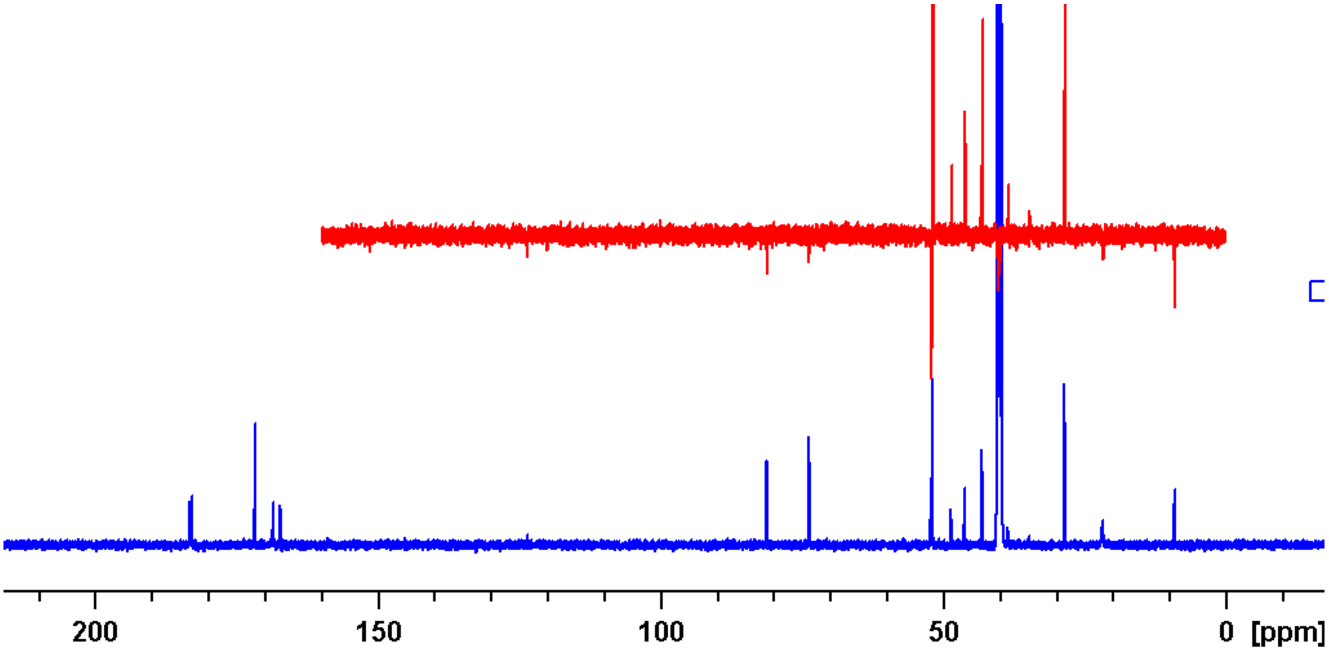

Figure S18. ^{13}C and DEPT NMR spectrum of the monomer **M** in DMSO-d_6

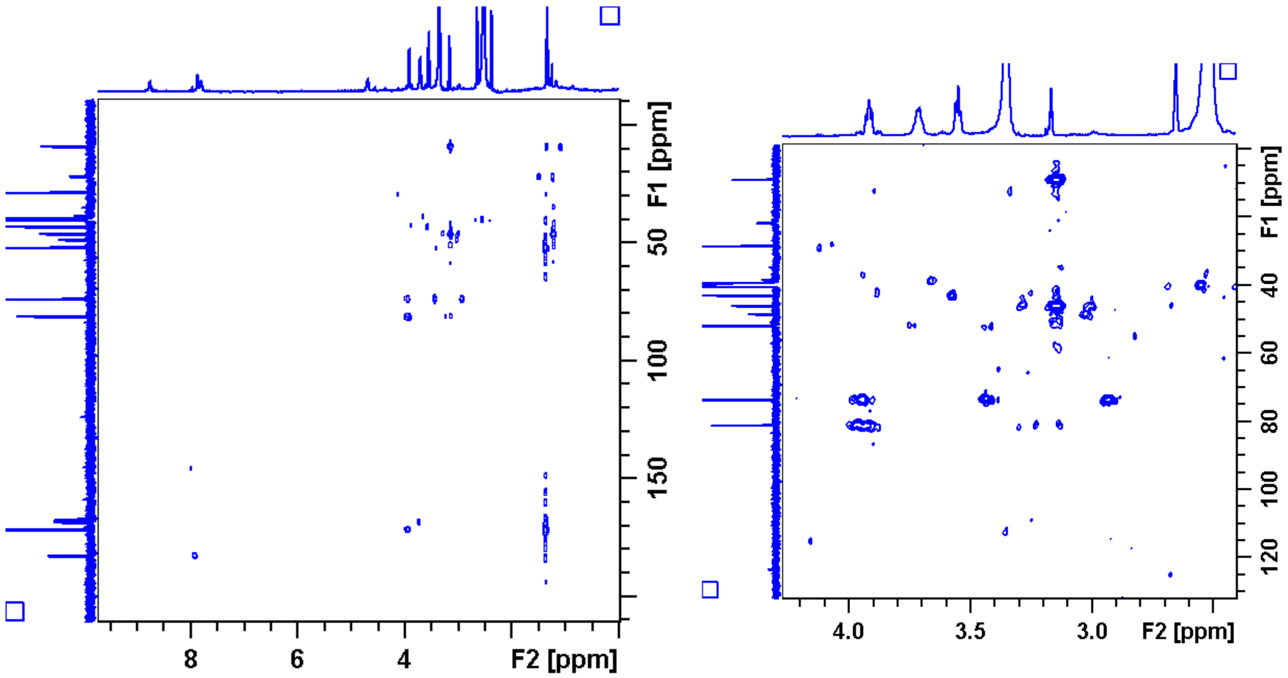

Figure S19. HMBC NMR spectrum of the monomer **M** in DMSO-d_6

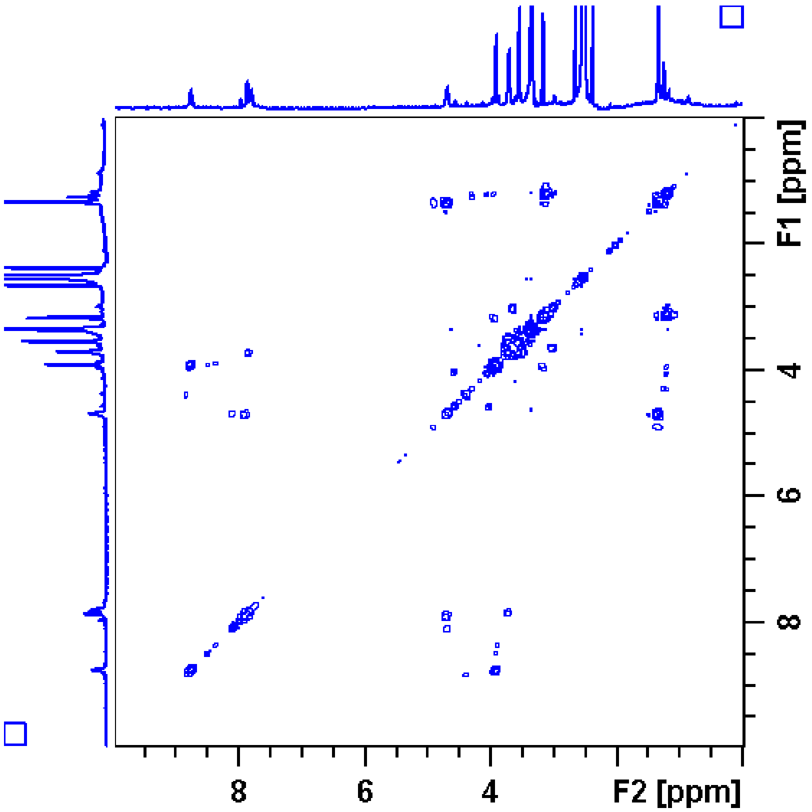

Figure S20. COSY NMR spectrum of the monomer **M** in DMSO-d$_6$

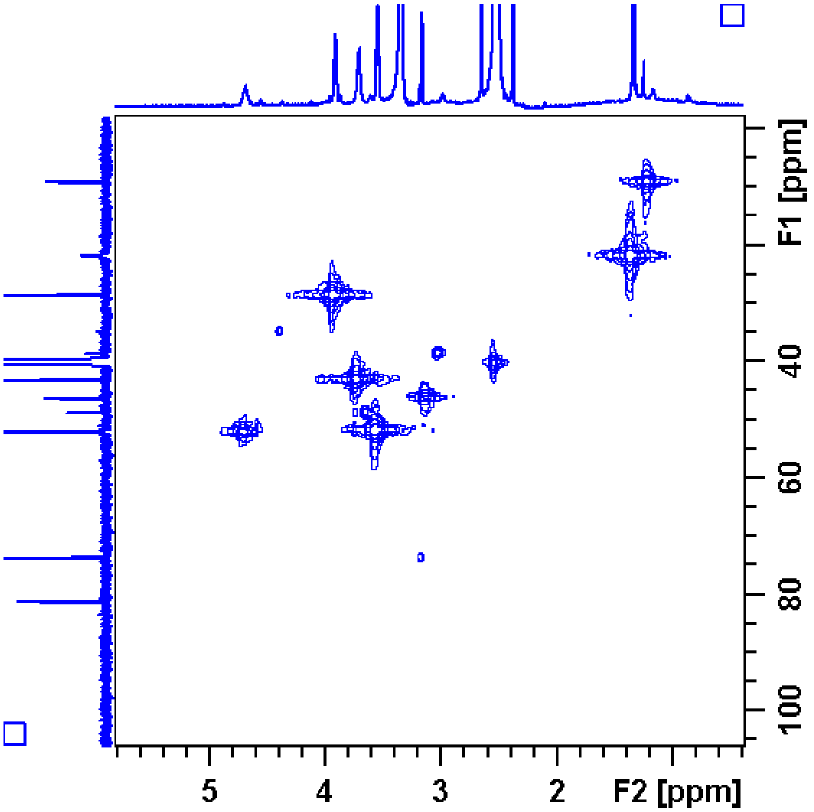

Figure S21. HMQC NMR spectrum of the monomer **M** in DMSO-d$_6$

22. References

1. Pathan, J. R.; Sureshan, K. M. Solvent-free and catalyst-free synthesis of crosslinkable polyfumaramides via topochemical azide-alkyne cycloaddition polymerization. *ACS Sust. Chem. Eng*. **2021**, *9*, 9871-9878.
2. Kondaparla, S.; Soni, A.; Manhas, A.; Srivastava, K. S. K.; Katti, B. Synthesis and antimalarial activity of new 4-aminoquinolines active against drug resistant strains. *RSC Adv*. **2016**, *6*, 105676-105689.
3. Diem, N. T.; Blaszkiewicz, C.; Menuel, S.; Roucoux, A.; Philippot, K.; Hapiot, F.; Monflier, F. Using click chemistry to access mono- and ditopic β-cyclodextrin hosts substituted by chiral amino acids. *Carbohydr. Res*. **2011**, *346*, 210-218.

23. Movie captions

Movie S1.
A crystal of **M** heated from 40 °C to 110 °C at a heating rate of 2 °C/min, shows smaller cracks.

Movie S2.
A crystal of **M** heated from 40 °C to 110 °C at a heating rate of 20 °C/min, shows wider and longer cracks.

Movie S3.
A crystal of **M** heated from 40 °C to 110 °C, at a heating rate of 1 °C/min, shows no cracks on the surface of the crystal.

Movie S4.
A crystal of **M** heated from 40 °C to 110 °C, at a heating rate of 3 °C/min, undergoes cracking and healing.

Movie S5.
A crystal of **M** heated from 40 °C to 110 °C, at a heating rate of 4 °C/min, shows cracking and healing.

Movie S6.
A crystal of **M** heated from 40 °C to 110 °C, at a heating rate of 5 °C/min, shows cracking and healing.

Movie S7.
A crystal of **M** heated from 40 °C to 110 °C, at a heating rate of 6 °C/min, showing cracking and healing.

Movie S8.
A crystal of **M** heated from 40 °C to 110 °C, at a heating rate of 7 °C/min, showing cracking and healing.

Movie S9.
A crystal of **M** heated from 40 °C to 110 °C, at a heating rate of 8 °C/min, showing cracking and healing.

Movie S10.
A crystal of **M** heated from 40 °C to 110 °C, at a heating rate of 9 °C/min, showing cracking and healing.

Movie S11.
A crystal of **M** heated from 40 °C to 110 °C, at a heating rate of 10 °C/min, showing cracking and healing.

Movie S12.

A crystal of **M** heated from 40 °C to 110 °C, at a heating rate of 12 °C/min, showing cracking and healing.

Movie S13.

A crystal of **M** heated from 40 °C to 110 °C, at a heating rate of 16 °C/min, showing cracking and healing.

Movie S14.

A crystal of **M** is heated from 50º C to 85 ºC at a heating rate of 13 ºC/s, held at 85 ºC for 3 s and then returned to 50 ºC. This heating and cooling cycle is repeated three times.

Movie S15.

A crystal of **M** is heated from 90 ºC to 120 ºC over 10 min. The crystal's shape changes as it reacts to form **P**. The video is played at 150x speed.
